# Nutrient starvation-induced Hda1C rewiring: coordinated regulation of transcription and translation

**DOI:** 10.1093/nar/gkaf256

**Published:** 2025-04-18

**Authors:** Min Kyung Lee, Byunghee Kang, Min-Kyung Shin, Yoon Ki Kim, Hye Young Kim, Soo Young Lee, Tae-Young Roh, TaeSoo Kim

**Affiliations:** Department of Life Sciences and Multitasking Macrophage Research Center, Ewha Womans University, Seoul 03760, Republic of Korea; Department of Life Sciences, Pohang University of Science and Technology (POSTECH), Pohang 37673, Republic of Korea; Department of Life Sciences, Ewha Womans University, Seoul 03760, Republic of Korea; Division of Life Sciences, Korea University, Seoul 02841, Republic of Korea; Department of Biological Sciences, Korea Advanced Institute of Science and Technology, Daejeon 34141, Republic of Korea; Department of Biomedical Sciences, Seoul National University College of Medicine, Seoul 03080, Republic of Korea; Department of Life Sciences and Multitasking Macrophage Research Center, Ewha Womans University, Seoul 03760, Republic of Korea; Department of Life Sciences, Ewha Womans University, Seoul 03760, Republic of Korea; Sysgenlab Inc., Pohang 37673, Republic of Korea; Department of Life Sciences and Multitasking Macrophage Research Center, Ewha Womans University, Seoul 03760, Republic of Korea

## Abstract

In yeast, Hda1 histone deacetylase complex (Hda1C) plays an important role in transcriptional regulation by modulating histone acetylation. We here explored the changes in Hda1C binding in nutrient-rich and -starved conditions. Chromatin immunoprecipitation sequencing revealed that starvation alters RNA Pol II and Hda1C binding to coding genes in a highly correlated manner. Interestingly, we discovered RNA Pol II transcription-independent recruitment of Hda1C to intergenic regions, particularly the upstream regulatory sequences (URS) of ribosomal protein (RP) genes, which are enriched with Rap1 binding sites. Under nutrient starvation, Rap1 contributes to the recruitment of Hda1C to these URS regions, where Hda1C deacetylates histones, thereby fine-tuning basal gene expression and delaying RP gene reactivation. Furthermore, Hda1C is also required for RNA Pol I transcription of ribosomal RNAs (rRNAs) and RNA Pol III transcription of transfer RNA (tRNA) genes, especially in nutrient-limited conditions. Significantly, Hda1C mutants are sensitive to translation inhibitors and display altered ribosome profiles. Thus, Hda1C may coordinate transcriptional regulation within the nucleus with translation control in the cytoplasm and could be a key regulator of gene expression responses to nutrient stress.

## Graphical Abstract

**Graphical Abstract:**
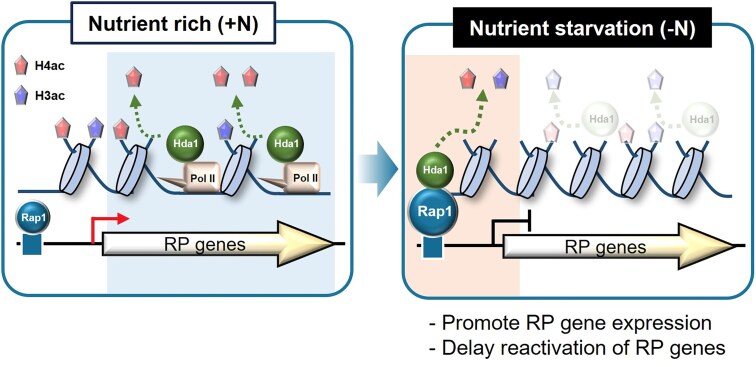

## Introduction

Post-translational modifications of histones include acetylation, methylation, phosphorylation, and ubiquitination [1]. All play pivotal regulatory roles in eukaryotic transcription. Histone acetylation is particularly important because it disrupts DNA-histone interactions and/or recruits factors that regulate chromatin structure, thereby directly activating RNA Polymerase II (RNA Pol II) transcription. Histone acetylation is dynamically regulated by the antagonistic functions of histone acetyltransferases (HATs) and histone deacetylases (HDACs) [2, 3]. Multiple HATs and HDACs are targeted to specific genomic regions by gene-specific transcription factors, by elongating RNA Pol II, or by recognizing co-transcriptional methylations [4, 5].

Hda1 histone deacetylase complex (Hda1C) is a class II HDAC identified in yeast [6]. It consists of a homodimer of the catalytic subunit Hda1 and a heterodimer of two non-catalytic subunits, Hda2 and Hda3 [7–9]. Hda1 contains a HDAC domain and an Arb2 domain at its N-terminus and C-terminus, respectively [10]. Both Hda2 and Hda3 have potential DNA-binding domains at their N-termini and coiled-coil domains at their C-termini [10]. The HDAC domain of Hda1 and the coiled-coil domains of both Hda2 and Hda3 are critical for the assembly and enzymatic activity of Hda1C [10]. *In vitro* studies show that the Arb2 domain of Hda1 directly interacts with nucleosomes and the DNA-binding domains of Hda2 and Hda3 can bind to single- or double-stranded DNA [9–11]. Moreover, recent *in vivo* studies showed that Hda1C preferentially binds to actively transcribed regions by interacting with elongating RNA Pol II, and that the Arb2 domain of Hda1 is crucial for this interaction [12, 13]. While the *in vivo* functions of the DNA-binding domains of both Hda2 and Hda3 remain unclear, several studies show that loss of Hda1C leads to global increases in the acetylation of histone H4 and H3 [6, 8]. An early study suggested that Hda1C represses transcription by deacetylating histone H3 and H2B at inactive promoters [14]. We recently found that Hda1C acts differently depending on transcription frequency. Specifically, while Hda1C indeed preferentially deacetylates histone H3 at inactive genes, it instead preferentially deacetylates histone H4 at actively transcribed genes. This H4 deacetylation at hyperactive genes likely affects RNA Pol II elongation since loss of Hda1C partially bypasses the requirement for the positive transcription elongation factor Bur1. By contrast, the H3 deacetylation at inactive genes appears to delay the kinetics of gene induction upon environmental changes. However, the mechanisms that determine the substrate specificity of Hda1C remain to be elucidated [12].

Transcription and translation are tightly coupled in prokaryotes: this facilitates efficient gene expression and rapid cellular responses to environmental changes. Although the research on such coupling in eukaryotes is much less extensive, there is some evidence of this coupling. Specifically, several nuclear factors have been found to influence translation. This generally involves them translocating from the nucleus to the cytoplasm. For example, in yeast, the Rpb4/7 subunit of RNA Pol II transiently moves to the cytoplasm to facilitate the export and stabilization of its target messenger RNAs (mRNAs) [15]. The yeast kinase Ctk1 and its homolog in higher eukaryotes, CDK12/13, may also relocate to the cytoplasm to influence translation. These kinases are involved in phosphorylating the C-terminal domain of Rpb1, the largest subunit of RNA Pol II, thereby promoting transcription [16]. However, they also bind to correctly processed messenger ribonucleoproteins (mRNPs) and travel with them to the cytoplasm, where they enhance efficient and accurate mRNA translation elongation [17]. Moreover, the well-known transcription factor p53 interacts with the cytoplasmic translation machinery, thereby promoting mRNA translation [18]. A recent study also showed that TAF7, a component of the TFIID complex, not only binds to target RNAs but also facilitates their export to the cytoplasm, thereby enhancing their translation [19]. Despite these findings, no evidence has yet demonstrated the existence of a single chromatin factor that simultaneously regulates transcription by RNA Pol I (for ribosomal RNAs [rRNAs]), RNA Pol II (for messenger RNAs [mRNAs]), and RNA Pol III (for transfer RNAs [tRNAs]), thereby coordinating transcription across different RNA polymerases with the process of translation.

Although it is established that Hda1C preferentially binds to hyperactive genes in a steady-state condition, it is not yet known whether this binding dynamically changes in response to gene induction or repression upon environmental shifts. Furthermore, whether Hda1C can be targeted to genomic regions outside of active coding regions remains unknown. In this study, we show that shifting yeast from nutrient-rich (+N) to nutrient-starvation (−N) conditions markedly changes the binding patterns of Hda1C and RNA Pol II in a highly correlated manner. Interestingly, beyond the coding regions, we found that starvation causes Hda1C to bind to many noncoding regions where RNA Pol II is absent. These regions include the upstream regulatory sequences (URS) of ribosomal protein (RP) genes. While Hda1C binds to the coding regions of active RP genes, nutrient starvation causes it to relocalize to the URS of the RP genes in the absence of RNA Pol II transcription. This relocalization is likely mediated by Hda1C interactions with Rap1 protein. Hda1C is needed for basal expression of RP genes in −N conditions and delayed activation of these genes upon nutrient resupply. Unexpectedly, Hda1C also binds to most tRNA genes and ribosomal DNA (rDNA) loci. In addition, deleting *HDA1* reduces RNA Pol III-mediated tRNA gene transcription and nascent pre-rRNA production by RNA Pol I in −N. Significantly, Hda1C is also required for normal growth in the presence of the translation inhibitors cycloheximide and hygromycin B in −N. Moreover, mutants for Hda1 display an abnormal pattern of ribosome profiling. Thus, Hda1C may contribute to efficient ribosome assembly and translation. These findings together suggest that Hda1C could serve as a nuclear factor that coordinates RNA Pol I, II, and III transcriptional activities in the nucleus and thereby shapes translation in the cytoplasm.

## Materials and methods

### Yeast strains and plasmids

Yeast strains used in this study are listed in Supplementary Table S1. The *HDA1*-myc, *RAP1*-myc, and *RPC40*-myc strains were generated by inserting the 18myc tag into the C-terminus of *HDA1*, *RAP1*, and *RPC40*, respectively. The previously described two-step tagging procedure was used for N-terminal AID (auxin-inducible degron) tagging of Rap1 with some modifications [20]. In the first step, the endogenous *RAP1* promoter (-377 to -1 relative to the +1 ATG start codon) was replaced with *TRP1* followed by the AID tag under the control of the *GAL1* promoter. The *TRP1*-p*GAL1* cassette and AID sequence were amplified with pFA6a-TRP1-pGAL1-3HA (Addgene plasmid #41609) and pKan-AID*-9myc (Addgene plasmid #99522), respectively by using primers that created Sal I sites. To construct *TRP1*-*PGAL1*-*AID*, these two polymerase chain reaction (PCR) products were ligated with the corresponding site. In the second step, the *TRP1* and *GAL1* promoter sequences were substituted with the natural *RAP1* promoter. The sequences of oligonucleotides used in this study are listed in Supplementary Table S2.

### Yeast culture conditions

Cells were grown in YPD [Yeast extract Peptone Dextrose: 1% Yeast extract, 2% Peptone, 2% Dextrose (Glucose)] at 30 °C until OD_600nm_ was 0.5–0.6, after which half were transferred to 0.15X YP (0.15% Yeast extract and 0.3% Peptone) for 4 h at 30 °C. For the time course experiment, wild-type and *hda1Δ* cells were grown overnight to OD_600nm_ 0.5–0.6 in 350 ml of YPD, transferred to 0.15X YP for 4 h at 30 °C, and then shifted back to YPD and grown at 22 °C for 1 h. The transcriptional dynamics were monitored at various time points (i.e. after overnight culture in YPD, after 4 h culture in 0.15X, and 8, 15, 30, and 60 min after the second YPD culture) by harvesting 50 ml of cells by centrifugation and analyzing their RNA. For Fig. 4F and Supplementary Fig. S5B–D, 3-indole-acetic acid (IAA; Sigma, I2886) was added to a final concentration of 0.5 mM in 0.15X YP media to induce Rap1 protein degradation in a starved condition. IAA stock (100 mM) was prepared in dimethyl sulfoxide (DMSO). The same volume of DMSO served as a control.

### Reverse transcription and qPCR analysis

RNA was extracted from cells with hot phenol. Total RNAs were treated with DNase I (Thermo Scientific Fisher) and first-strand complementary DNA (cDNA) synthesis was performed with 1 μg total RNA using ReverTra Ace^™^ qPCR RT kit (TOYOBO) and gene-specific primers. cDNA was analyzed by real-time quantitative PCR (qPCR) using THUNDERBIRD SYBR qPCR Mix (TOYOBO) and CFX96 cycler (Bio-Rad) [21].

### Chromatin immunoprecipitation and ChIP-sequencing

Chromatin immunoprecipitation (ChIP) was performed as previously described [12, 22] with oligonucleotides listed in Supplementary Table S2. The following antibodies were bound to either Protein G or Protein A agarose beads overnight: anti-Rpb3 (BioLegend, 665004), anti-H3 (Abcam, Ab1791), anti-H4ac (Millipore, 06-598), anti-H3ac (Millipore, 06-599), and anti-myc (BioLegend, 626802). Binding for anti-Rpb3, anti-H3, and anti-myc was done in FA lysis buffer containing 275 mM NaCl. For anti-H4ac and anti-H3ac, FA lysis buffer containing 1 M NaCl was used for antibody binding. Precipitates were washed once with the same buffer; then once with 500 mM NaCl-containing FA lysis buffer (for anti-Rpb3, anti-H3, and anti-myc) or 1.5 M NaCl-containing FA lysis buffer (for anti-H4ac and anti-H3ac); once with TE [10 mM Tris–HCl (pH 8.0) and 1 mM EDTA] containing 0.25 M LiCl, 0.5% NP-40, 0.5% sodium deoxycholate; and once with TE. The precipitated DNAs were analyzed by qPCR using THUNDERBIRD SYBR qPCR Mix (TOYOBO) and CFX96 cycler (Bio-Rad). *Schizosaccharomyces pombe* “spike-in” chromatin was added at 10% relative to *Saccharomyces cerevisiae* chromatin for ChIP-seq samples, and normalization was carried out except for H3 acetylation.

ChIP-sequencing (ChIP-seq) libraries were constructed using the Accel-NGS 2S Plus DNA Library Kit (Swift BioSciences, 21096) according to the manufacturer’s instructions. Briefly, purified immunoprecipitated DNA was subjected to end-repairing and adapter ligation and amplified by 12 cycles of PCR. The amplified DNA was size-selected and quantified using Bioanalyzer (Agilent). The ChIP-seq libraries were sequenced on an HiSeq2500 platform (Illumina).

### ChIP-seq data analysis

Sequence quality was examined by FastQC (version 0.11.5) (https://www.bioinformatics.babraham.ac.uk/projects/fastqc/). Illumina adapters were trimmed by cutadapt (version 1.18) [23]. All reads were aligned to the merged reference composed of *S. cerevisiae* genome (R64-1-1) and *S. pombe* genome (ASM294v2) by using Bowtie2 program (version 2.3.4.1) [24]. Reads that were exclusively assigned to each genome were counted to remove ambiguity. Low-quality reads (PCR duplicates, unmapped, or mate unmapped reads, not primary alignment, and reads failing platform) were excluded by SAMtools (version 1.7) [25] and Picard-tools (version 2.22.9) (https://broadinstitute.github.io/picard/). MACS2 (version 2.1.1) [26] was used to call peaks for each sample with parameters “-q 0.01”. Normalization with the *S. pombe* spike-in control was conducted as previously described [12]. Briefly, the ratio of the read numbers from the treated samples to the read numbers from the control samples was used with MACS2 parameter “–ratio”. DeepTools (version 3.1.3) [27] was used to draw heatmaps of read enrichment and to calculate read counts on the peak and CDS regions. Differential Rpb3-binding analysis was performed by using R package DESeq2 (version 1.30.1) [28]. Robust exponential regression analysis of Hda1 binding on Rpb3 binding was conducted with R package robustbase (version 0.95-0) (http://robustbase.r-forge.r-project.org). The bins (200 bp) whose residual was greater than 2.576 were considered to be outliers. Before deciding Pol II-associated/independent Hda1-binding regions, the Hda1 peak was divided into Rpb3 peak-overlapping and non-overlapping peaks (peaks shorter than 100 bp were excluded). GO-term enrichment analysis was performed using YeastEnrichr [29]. TF-binding motif enrichment and Rap1-binding motif local enrichment analysis were conducted with AME [30] and CentriMo [23] from MEME-suite (version 5.4.0).

### Co-immunoprecipitation analysis

Whole cell extracts were prepared with binding buffer [50 mM Tris–HCl (pH 7.5), 0.1% NP-40] containing 150 mM NaCl and protease inhibitors [Pepstain A, 1 μM; Aprotinin, 0.3 μM; Leupeptin, 1 μM; Phenylmethylsulfonyl fluoride (PMSF), 1 mM; and *N*-ethylmaleimide, 25 mM]. Five milligrams of total extracts were incubated with 20μl of Protein G or Protein A bead (Cytiva) and with or without 8μl anti-myc (BioLegend) at 4 °C overnight. The beads were washed five times with 1 ml of binding buffer, and the precipitates were resolved by SDS–PAGE followed by immunoblot analysis.

### Western blot analysis

Cells were lysed with lysis buffer [50 mM Tris (pH 7.5), 150 mM NaCl, and 0.1% NP-40] containing protease inhibitors (Pepstain A, 1 μM; Aprotinin, 0.3 μM; Leupeptin, 1 μM; PMSF, 1 mM; and *N*-ethylmaleimide, 25 mM) and glass beads. Protein concentration was quantitated by Bradford assay. For SDS–PAGE and western blot analyses, 20–90 μg of whole cell extracts were used. Proteins were separated by SDS–PAGE and transferred to a nitrocellulose-blotting membrane (Cytiva). The membranes were incubated with primary antibody and then washed with PBST. After incubation with HRP-conjugated secondary antibody, the membranes were washed three times with PBST. The blots were visualized on film (AGFA) with Super Signal West Pico PLUS Chemiluminescent Substrate (Thermo Fisher Scientific). Anti-Rap1 (Santa Cruz, sc-374297, sc-166556), anti-myc (BioLegend, 626802), and anti-Rpb3 (BioLegend, 665004) were used for western blot analysis.

### Spot assay

For spotting analysis, cells were resuspended and diluted to OD_600_ = 1.0 and subjected to 3-fold serial dilutions. Two microliters of aliquots of each dilution were spotted on YPD, 0.15X YP, or SC (Synthetic Complete) plate in the presence or absence of transcription or translation inhibitors.

### Polysome profiling

WT and *hda1Δ* cells were grown overnight to OD_600nm_ 0.5–0.6 in 80 ml of YPD medium at 30 °C, after which half (40 ml of cells) were subsequently transferred to 0.15X YP medium for 4 h at 30 °C. Forty milliliters of cells were treated with 50 μg/ml cycloheximide for 15 min at 30 °C before harvesting by centrifugation. Pelleted cells were washed with 2 ml of ice-cold lysis buffer [20 mM Tris–HCl (pH 7.5), 8 mM MgCl_2_, and 100 mM KCl] with freshly added 12 mM β-mercaptoethanol, 200 μg/ml cycloheximide, and 1 mM PMSF. Cells were lysed with 0.5 mm glass beads and 350 μl ice-cold lysis buffer with freshly added β-mercaptoethanol, cycloheximide, and PMSF by vortexing for 30 sec and then cooling down on ice for 1 min, for a total of eight cycles. Whole cell extracts were loaded onto a 10%–50% sucrose gradient and subjected to ultracentrifugation at 36,000 rpm for 2 h (Optima XE-90 Ultracentrifuge, Beckman Coulter) with the following settings: rapid acceleration followed by gradual deceleration. Each fraction was collected using a Foxy R1 Fraction Collector (Teledyne ISCO) according to the *A*_254_ absorbance.

## Results

### Dynamic changes of Hda1C binding to coding regions upon nutrient starvation

**Figure 1.**
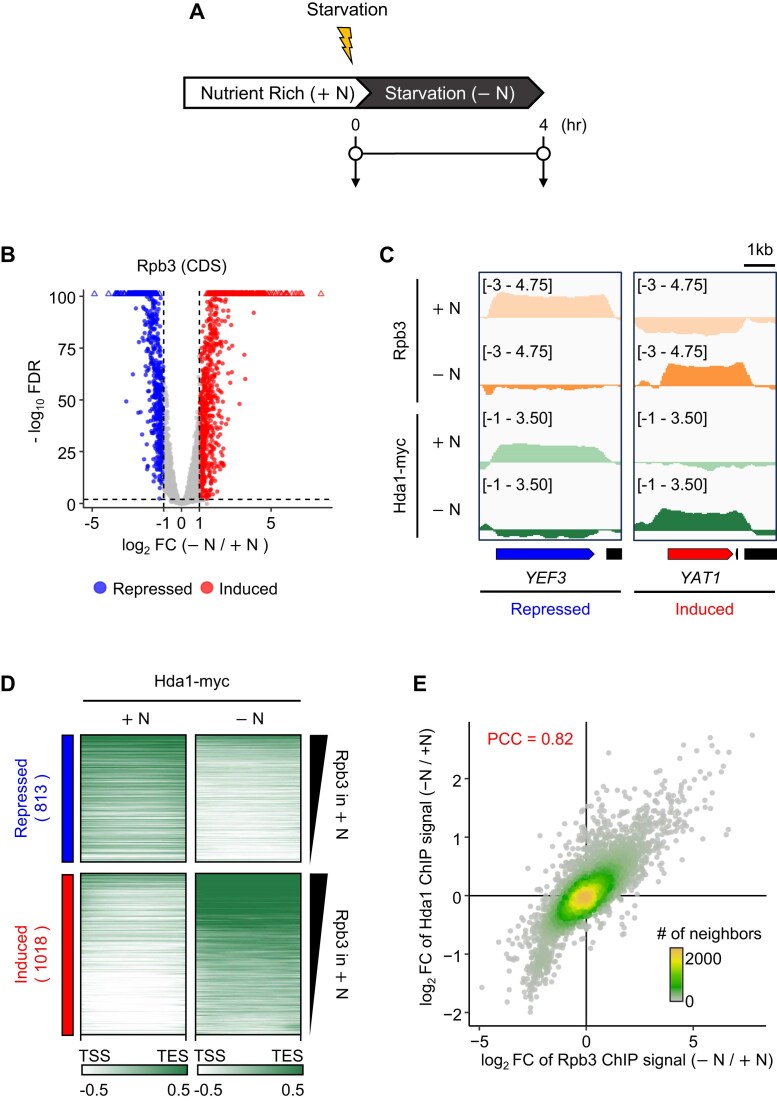
Binding of Hda1C to coding regions correlates with transcription frequency. (**A**) Schematic representation of the nutrient starvation conditions (+N → −N) used to assess the effect of nutritional changes on transcriptional reprogramming. Nutrient-rich = YPD medium = +N; Starvation = 0.15X YP medium = −N. (**B**) Volcano plot of differential Rpb3 (RNA Pol II) binding to mRNA genes, based on the average of two independent ChIP-seqs of wild type cells that were subjected to +N and −N. *S. pombe* chromatin was included as spike-in controls. Two-fold change and 0.01 FDR thresholds were considered significant. The 813 downregulated and 1018 upregulated genes under -N conditions are labeled as "repressed" and "induced" in the plot. (**C**) ChIP-seq tracks of the Rpb3 and Hda1-myc ChIP signals at *YEF3* (a gene that is repressed by −N) and *YAT1* (a gene that is induced by −N), based on the average of two independent ChIP-seqs of wild type cells that were subjected to +N and −N. *S. pombe* chromatin was included as spike-in controls. (**D**) Heatmaps of Hda1-myc ChIP signals at the 813 and 1018 genes that are repressed and induced by −N, respectively. The average of the two independent experiments including *S. pombe* spike-in controls is shown. The genes are sorted in descending order of Rpb3 level in ${\mathrm{ + }}$N conditions. The *y*-axis indicates each gene, and the *x*-axis indicates relative position to TSS and transcription end site (TES). (**E**) Changes of Hda1 binding and Rpb3 occupancy at coding regions show a strong positive correlation. Differential Hda1-myc occupancy in −N compared to +N was plotted against differential Rpb3 occupancy in +N and −N. Pearson’s correlation coefficient is indicated (PCC ${\mathrm{ = \ }}$0.82).

We showed previously that Hda1C preferentially binds to hyperactive coding regions in steady-state conditions [12], but it is unclear whether this preference is maintained when cells are exposed to environmental changes that alter gene expression. To test this, cells were grown in nutrient-rich medium (YPD; designated +N) and then shifted to nutrient-depleted medium (0.15X YP; designated −N) for 4 h (Fig. 1A). We first examined the effect of starvation on the transcription of coding genes, as indicated by RNA Pol II (Rpb3) binding. Chromatin immunoprecipitation (ChIP) qPCR analyses of two typical genes that are active in +N, *YEF3* and *PMA1*, showed that while RNA Pol II associated strongly with their coding regions in +N, starvation abolished this binding (Supplementary Fig. S1A). ChIP-sequencing (ChIP-seq) analyses using *S. pombe* spike in controls as previously described [12] then showed that starvation changed RNA Pol II binding at a genome-wide level: application of 2-fold change and 0.01 false discovery rate (FDR) thresholds indicated that starvation repressed and induced transcription of 813 and 1018 genes, respectively (Fig. 1B and Supplementary Fig. S1B). These data also confirmed that −N abolished the strong binding of RNA Pol II to *YEF3* that was observed in +N (Fig. 1C). Conversely, analysis of *YAT1*, a gene that is induced by starvation, showed that RNA Pol II bound poorly to it in +N, but this binding was greatly increased by starvation (Fig. 1C). We next used the ChIP-seq data to determine the Hda1 binding patterns. We found that they closely mirrored the Rpb3 binding patterns: the 1018 genes that were switched on by starvation (e.g. *YAT1*) had high Hda1 levels while the 813 genes that were switched off by starvation (e.g. *YEF3*) exhibited less Hda1 binding (Fig. 1C and D, and Supplementary Fig. S1C). Additionally, a strong positive correlation between changes in Hda1 distribution and RNA Pol II binding was observed (Fig. 1E). Under these conditions, Hda1 protein levels remained unchanged (Supplementary Fig. S1D). Collectively, these findings indicate that Hda1C-binding pattern at coding regions dynamically changes depending on the transcriptional activity of each gene.

**Figure 2.**
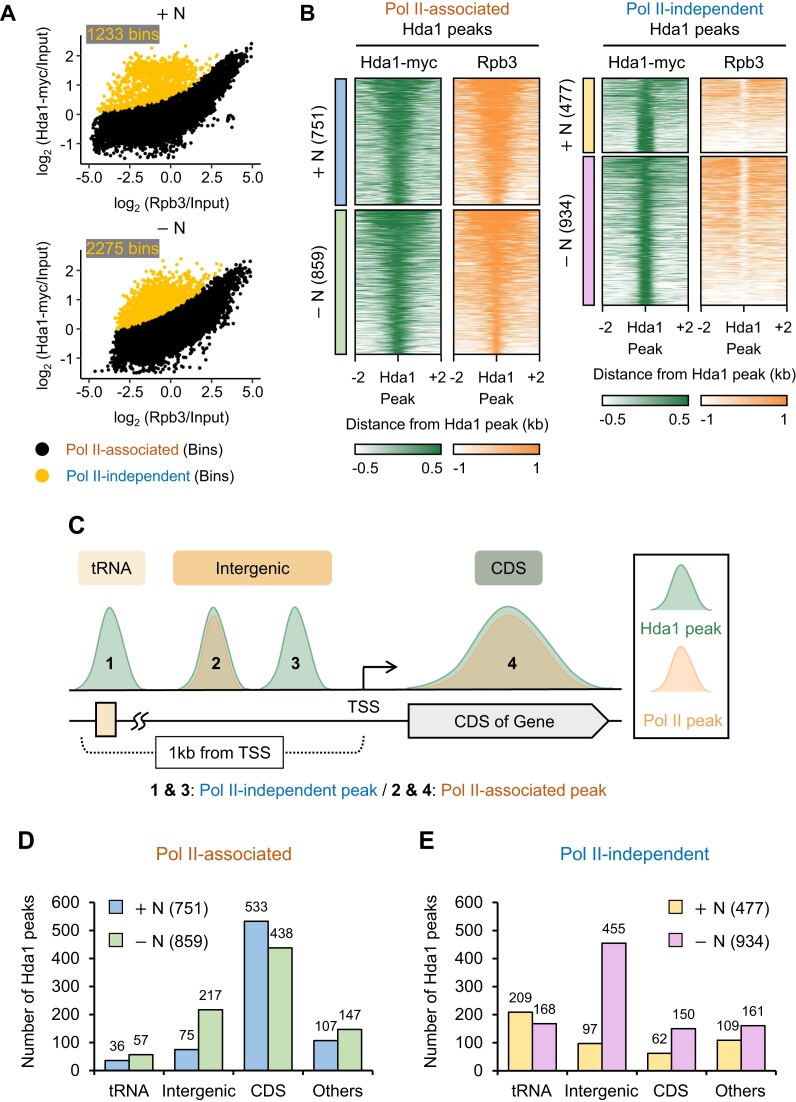
RNA Pol II-independent targeting of Hda1C. (**A**) Hda1C can bind to the genome in a transcription-independent manner in +N and −N conditions. The average occupancy of Hda1-myc from two-independent ChIP-seqs including *S. pombe* chromatin as spike-in controls was plotted against Rpb3 level in +N and −N conditions, respectively. For this, the entire genome except the 200 bp end region of each chromosome was divided into 60333 200 bp bins. A robust nonlinear regression model revealed Pol II-associated bins (fitted bins, black) and Pol II-independent bins (outlier bins, yellow) in both conditions. The numbers of Pol II-independent bins are indicated. (**B**) Hda1 peaks in +N and −N were subdivided into Pol II-associated (+N: *n* = 751, blue; −N: *n* = 859, green) and Pol II-independent peaks (+N: *n* = 477, yellow; −N: *n* = 934, pink) based on whether they overlapped with Rpb3 peaks. Heatmaps of the ChIP-seq signals for Hda1-myc and Rpb3 around Hda1 peaks are shown. The signals were plotted over 4 kb windows centered on the Hda1 peaks. Each genomic region is presented in descending order according to Rpb3 levels. (**C**) Schematic representation depiction of the major regions that bear Hda1 peaks in +N/−N conditions. The Hda1 peaks were classified into four groups based on their genomic location: (i) tRNA, (ii) and (iii) Intergenic, (iv) CDS, and others. (**D** and **E**) The number of Pol II-associated (D) and Pol II-independent (E) Hda1 peaks in the different genomic regions. Most Pol II-associated Hda1 peaks were found at CDS in both +N and −N whereas most Pol II-independent Hda1 peaks were at tRNAs in +N and at Intergenic regions −N.

Hda1C preferentially deacetylates histone H4 at active genes and histone H3 at inactive genes in steady-state conditions [12]. To determine whether this pattern is also observed in starvation conditions, wild type and *hda1Δ* cells were grown in +N and −N and subjected to ChIP-seq using antibodies that recognize tetra-acetyl H4 (H4ac) or di-acetyl H3 (H3ac). The acetylation levels were normalized to total histone content, as measured by H3 levels. We first examined the 813 genes repressed by −N in wild type cells, which are relatively active in +N. In this condition, loss of Hda1 increased H4 acetylation but not H3 acetylation at these genes. By contrast, in −N, which repressed these genes, loss of Hda1 substantially increased H3 acetylation, while H4 acetylation remained largely unchanged, with a slight increase likely due to the incorporation of pre-existing acetylated histones (Supplementary Fig. S1E). The converse pattern was observed for the 1018 genes that are induced by −N (Supplementary Fig. S1F and G). These findings suggest that Hda1C differentially deacetylates histones depending on the transcriptional status of each gene, preferentially targeting H4 in transcriptionally active states and H3 in repressed states.

### Starvation induces transcription-independent Hda1C binding at intergenic regions

We next examined the RNA Pol II-dependency of the binding of Hda1C to other areas of the genome and the effect of starvation on this. For this, the genome was subdivided into 60333 200 bp bins. We detected bins that concomitantly showed low RNA Pol II ChIP signals and high Hda1 ChIP signals (termed Pol II-independent bins) in both +N and −N (Fig. 2A). Notably, the RNA Pol II-independent bins were twice as common in the starved condition: +N and −N associated with 1233 and 2275 RNA Pol II-independent bins, respectively. By contrast, starvation did not significantly change the number of bins that had both RNA Pol II and Hda1 (termed Pol II-associated bins) (Supplementary Fig. S2A). We obtained similar results when we classified the Hda1 ChIP-seq peaks in +N and −N on the basis of whether they did/did not overlap with RNA Pol II ChIP-seq peaks: there were 751 and 859 Pol II-associated Hda1 peaks in the +N and −N conditions, respectively, and 477 and 934 Pol II-independent Hda1 peaks in the +N and −N conditions, respectively (Fig. 2B). In contrast, nutrient deprivation doubled both the Pol II-independent bins (from 1233 to 2275) and Hda1 peaks (from 477 to 934) while having little effect on the number of Pol II-associated bins and Hda1 peaks (Fig. 2B and Supplementary Fig. S2A). These findings show that Hda1C can also bind to the genome independently of RNA Pol II transcription, and that this pattern becomes 2-fold more common when the cells are starved.

**Figure 3.**
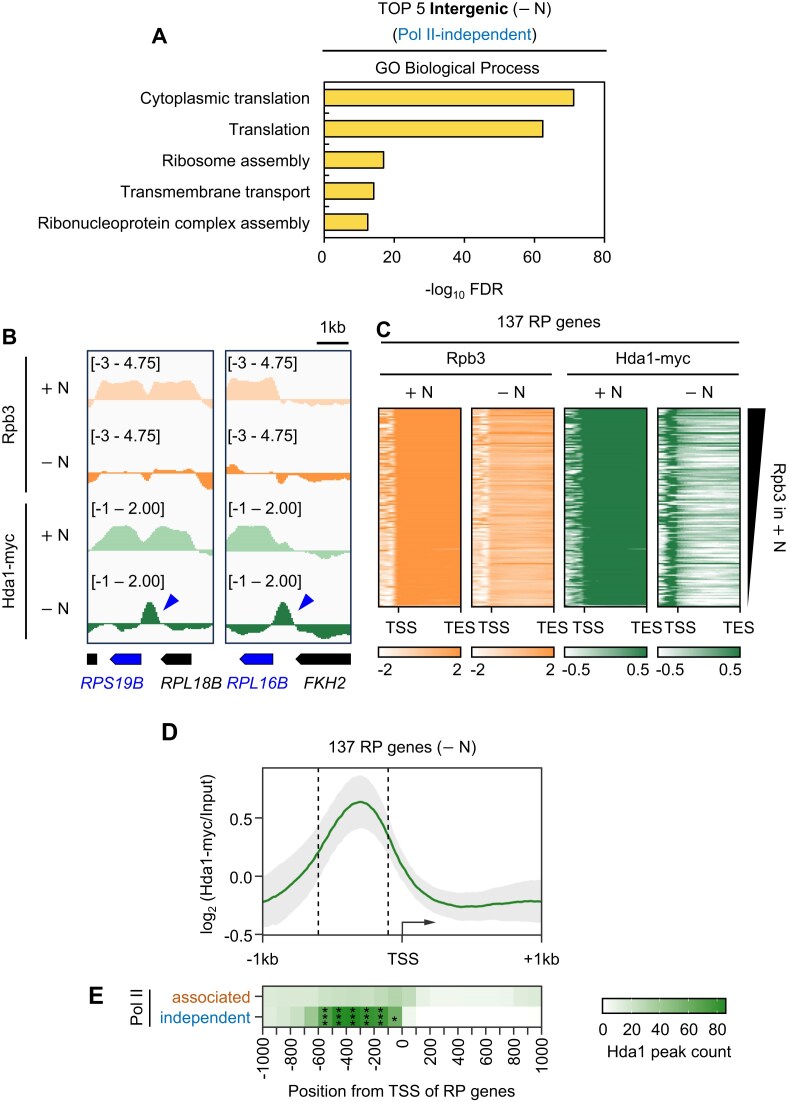
RNA Pol II-independent targeting of Hda1C to the URS of RP genes. (**A**) Gene ontology (GO) analysis of enriched biological processes for mRNA genes where Pol II-independent “Intergenic” −N Hda1 peaks are found within 1 kb upstream region. GO terms associated with translation (GO:0002181 and GO:0006412) were enriched at these genes. (**B**) ChIP-seq tracks of the Rpb3 and Hda1-myc ChIP signals at *RPS19B* and *RPL16B* in +N and −N conditions. (**C**) Starvation induces Hda1C to relocalize from the coding regions of RP genes to their URS. Heatmaps of Rpb3 and Hda1-myc ChIP signals at all 137 RP genes in +N and −N. Genes are sorted in descending order of Rpb3 levels in +N (*y*-axis). The *x*-axis indicates the position relative to the 1 kb upstream region, the TSS, and the TES. (**D**) Line plot showing the average Hda1-myc ChIP-seq signals in the 1 kb region flanking the TSS of the 137 RP genes in −N conditions. Standard deviation (S.D.) was indicated in gray. (**E**) Heatmap showing that the RNA Pol II-independent Hda1 peaks in −N are particularly enriched in the −600 bp to −100 bp region of the 137 RP genes. Statistical significance was calculated using the Poisson test (*FDR ${\mathrm{ < }}$ 0.05, and ***FDR ${\mathrm{ < }}$ 0.001).

Interestingly, the Pol II-independent Hda1 peaks were narrower than the Pol II-associated Hda1 peaks (Fig. 2B). Each Pol II-independent Hda1 peak included ∼3 bins (Supplementary Fig. S2A). Thus, Pol II-independent Hda1 peaks have distinct features compared to Pol II-associated peaks. We therefore investigated the frequencies of Pol II-associated and Pol II-independent Hda1 peaks in four functional genomic units termed tRNA, Intergenic, Coding Sequences (CDS), and Others (Fig. 2C). Note that we separated the tRNA regions from the Intergenic regions because Hda1C has been shown previously to bind at tRNA loci [31]. Our Hda1 peak classification process was as follows. First, we identified all Hda1 peaks that included an entire tRNA gene: these were considered to be in the tRNA genomic unit. Second, we identified the Hda1 peaks that covered at least 30% of a coding region: these were allocated to the CDS genomic unit. Third, we subdivided the 1 kb upstream region of the transcription start sites (TSS) into five 200 bp subregions and identified the Hda1 peaks that covered 90% of any of these five subregions. If neither tRNA nor the CDS of other genes were present in the upstream subregion(s), these Hda1 peaks were assigned to the Intergenic genomic unit. Finally, peaks that did not satisfy any of these criteria were categorized as others (Fig. 2C).

With regard to the Pol II-associated Hda1 peaks, most were in the CDS genomic unit regardless of the nutrition condition. Specifically, in +N and −N, 71% (533/751) and 51% (438/859) of the Pol II-associated Hda1 peaks were in the CDS unit, respectively (Fig. 2D). Gene ontology (GO) analysis of the genes with Hda1 peaks showed that they differed depending on the nutrition conditions: in +N, they related strongly to translation, whereas in −N, they related predominantly to respiration (Supplementary Fig. S2B and C). Two examples of Pol II-associated Hda1 peaks in the Intergenic unit are shown in Supplementary Fig. S2D: one is the small nuclear RNA gene *snR64* and the other is an ncRNA region. Both are transcribed by RNA Pol II. By contrast, few of the Pol II-independent Hda1 peaks were in the CDS unit: in +N and −N, only 13% (62/477) and 16% (150/934) of these peaks covered coding genes, respectively (Fig. 2E). As Hda1C was previously known to bind at tRNA loci [31], the Pol II-independent Hda1 peaks also overlapped with tRNA genes. Examples of Pol II-independent Hda1 peaks at tRNAs [*tV(AAC)O* and *tH(GUG)G2*] are shown in Supplementary Fig. S3A. Importantly, the Pol II-independent Hda1 peaks were concentrated in the Intergenic unit, particularly in the −N condition (Supplementary Fig. S3B): in −N, 48% (455/934) of these peaks were categorized as Intergenic versus 20% (97/477) of the peaks in +N (Fig. 2E). This represents a 4.7-fold starvation-induced increase. In contrast, Pol II-associated Hda1 peaks were predominantly in CDS regions regardless of nutrition status. Starvation increased particularly Pol II-independent Hda1 binding at intergenic regions. These data together show that starvation not only doubles Pol II-independent Hda1C binding to the genome, but much of this increased binding occurs at intergenic regions.

### Starvation induces Hda1C to relocalize from the CDS of RP genes to their URS

To assess the transcription-independent binding of Hda1C to intergenic regions more closely, we identified all mRNA genes that exhibited Pol II-independent Intergenic Hda1 peaks in their 1 kb upstream region in −N conditions. GO analysis of these genes showed that “cytoplasmic translation” and “translation” were the most enriched categories (Fig. 3A). Since 86% of the genes within these terms are ribosomal protein (RP) genes, we examined the transcription and Hda1 binding of the 137 RP genes in the yeast genome more closely.

Under optimal growth conditions, RP genes are actively transcribed and constitute ∼50% of all RNA Pol II-mediated transcription. However, stress significantly represses RP gene expression [32, 33]. Our ChIP-seq analysis of the 137 RP genes in wild type in +N and −N also showed this. Examples are shown in Fig. 3B: the coding regions of *RPL16B* and *RPS19B* demonstrated high RNA Pol II occupancy in +N, and this was completely abrogated in −N. Interestingly, this starvation-induced loss of transcription was not only matched by ablation of the strong Hda1 binding to these coding regions, but it also associated with new RNA Pol II-independent binding of Hda1 to the upstream intergenic regions of these genes (blue arrowheads in Fig. 3B). This novel starvation-induced relocalization of Hda1 from a coding region to its upstream intergenic region was observed globally across RP genes (Fig. 3C, right). While the average ChIP-seq Hda1 signals at all 137 RP genes under +N conditions showed strong enrichment in coding regions (Supplementary Fig. S4), a line plot of the average signals in −N conditions revealed that the relocalized Pol II-independent Hda1 peaks were specifically enriched in the region from 600 to 100 bp upstream of the RP genes, overlapping with the upstream regulatory sequence (URS) of RP genes (Fig. 3D and E). Thus, starvation induces Hda1C to relocalize from the coding region to the URS of the same gene. These findings indicate that Hda1C may regulate the expression of key translation-related genes upon nutrient starvation.

**Figure 4.**
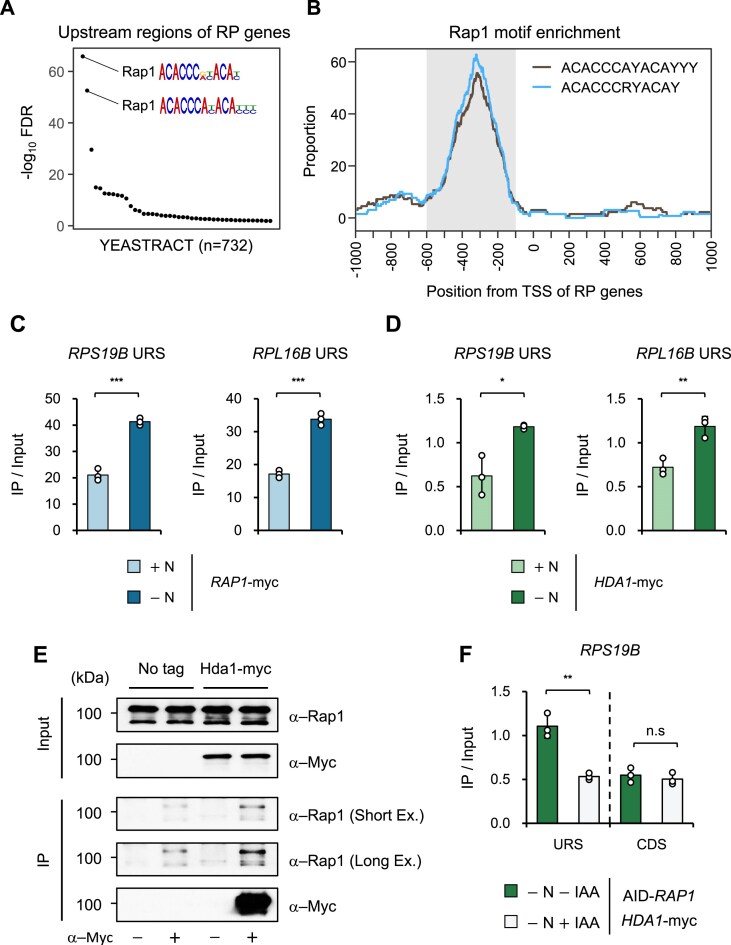
Rap1 is required for the recruitment of Hda1C to the URS of RP genes. (**A**) Two Rap1-binding motifs (ACACCCAYACAYYY and ACACCCRYACAY) are enriched in the 1 kb upstream region of the 137 RP genes. (**B**) Location of the two Rap1-binding motifs in the 1 kb regions that flank the TSS of the 137 RP genes. RNA Pol II independent Hda1 peaks in −N identified in Fig. 3E were indicated in gray. (**C** and **D**) The binding of Rap1 and Hda1 to the URS of RP genes is increased upon starvation. Occupancy of Rap1-myc and Hda1-myc in +N and −N conditions was monitored by ChIP assay using the indicated strains. Crosslinked chromatin was precipitated with anti-myc antibody. qPCR analysis of the precipitated DNA was carried out on the URS of *RPS19B* and *RPL16B*. The signals for Rap1-myc and Hda1-myc were quantitated and normalized to the input signal. A nontranscribed region near the telomere of chromosome VI was used as an internal control for ChIP assay using *HDA1*-myc. Error bars show the standard deviation (S.D.) calculated from three biological replicates, each with three technical replicates. *P${\mathrm{ < }}$ 0.05, **P${\mathrm{ < }}$ 0.01, and ***P$ <$ 0.001 (two-tailed unpaired Student’s *t* tests). (**E**) Hda1 physically interacts with Rap1 in −N. Whole cell extracts from the indicated strains were incubated with anti-myc antibody and protein G beads. The precipitates (IP) were analyzed by immunoblotting for myc-tagged proteins (Hda1-myc) and Rap1. (**F**) Hda1C binding to the URS of RP genes is reduced upon depletion of Rap1 in −N conditions. *HDA1*-myc cells expressing AID-Rap1 were grown in +N and then shifted to IAA containing −N medium (−N + IAA) for 4 h to deplete Rap1. An equal volume of DMSO in −N (−N–IAA) served as a negative control. ChIP-qPCR analysis of *RPS19B* was performed using anti-myc antibody as in Fig. 4D.

### Rap1 is required for Pol II-independent Hda1 binding to the URS of RP genes

We next asked whether transcriptional activators or repressors that bind to specific DNA sequences help recruit Hda1 to the URS of RP genes. To identify possible candidates, we analyzed the DNA sequence motifs in the 1 kb upstream regions of the 137 RP genes. The top-ranking motifs were two consensus sequences for Repressor activator protein 1 (Rap1) (Fig. 4A). Both sequences were also specifically detected in the URS regions of the RP genes that Hda1 bound in starvation conditions (Fig. 4B).

Rap1 binds constitutively to the URS of RP genes regardless of their transcriptional status [34–36]. We confirmed this with a ChIP assay using Rap1-myc cells. Interestingly, while Rap1 bound to RP gene URS in both +N and −N conditions, it was doubled by starvation mirroring the pattern of Hda1 binding (Fig. 4C and D). These data suggest that Rap1 could mediate starvation-induced recruitment of Hda1 to the URS of RP genes.

To test this, we first examined the physical interaction between Hda1 and Rap1 in −N conditions by co-immunoprecipitation assay. This revealed a weak but significant interaction between the two proteins (Fig. 4E and Supplementary Fig. S5A). Next, we asked whether Rap1 is directly needed for Hda1 targeting by depleting Rap1 using the AID system [37]. Thus, cells expressing N-terminal AID-tagged Rap1 were grown in +N medium and then transferred to IAA (i.e. auxin)-containing −N medium for 4 h. The auxin treatment reduced the Rap1 protein levels of the starved cells by ∼94% but had no effect on Hda1 levels (Supplementary Fig. S5B and C). This depletion of Rap1 significantly decreased Hda1 binding to the URS of *RPL16B*, *RPS19B*, and *RPL24A* but did not change Hda1 binding at the coding region (Fig. 4F and Supplementary Fig. S5D). Thus, Rap1 physically interacts with Hda1 and contributes to Hda1 targeting to the URS of RP genes in −N conditions.

### Histone deacetylation by Hda1C at the URS affects RP gene expression

**Figure 5.**
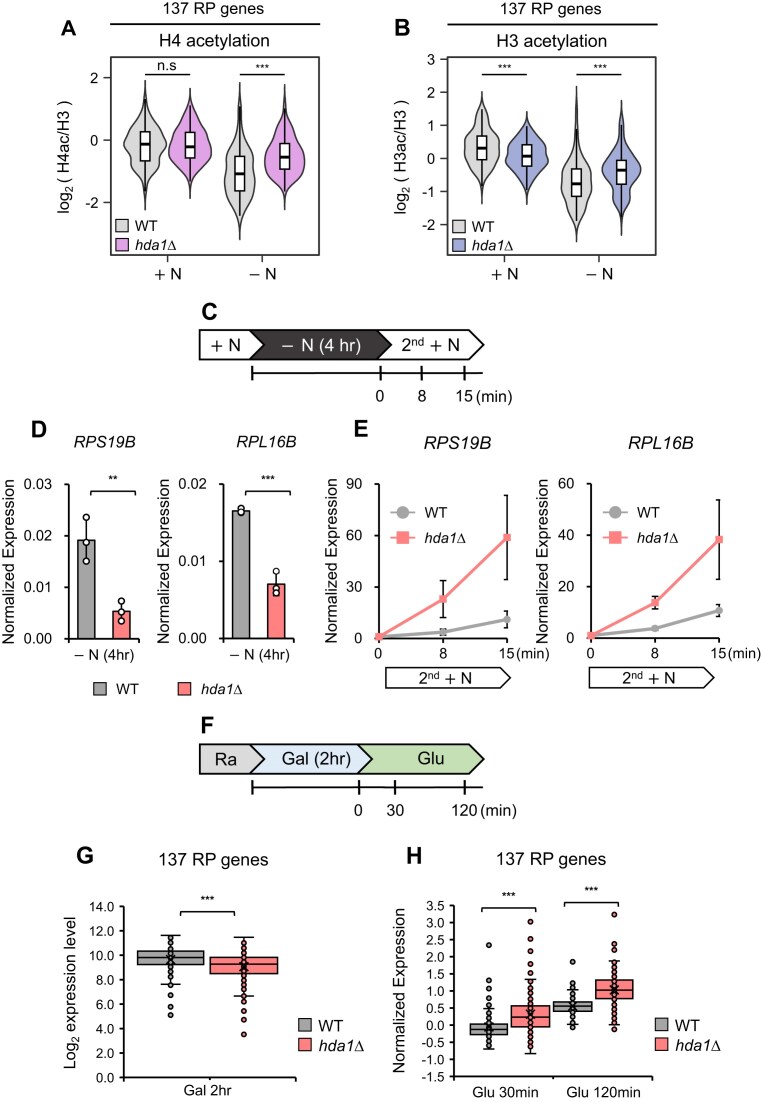
Histone deacetylation by Hda1C at the URS affects RP gene expression. (**A** and **B**) Hda1C deacetylates histone H3 and H4 at the URS of RP genes in −N. The violin plot shows the average H3 and H4 acetylation levels at the −600 to −100 bp region of all 137 RP genes in WT and *HDA1*-deleting cells. The H4 acetylation and H3 acetylation levels were normalized to total H3 content and calculated in log_2_ ratio. The data are from two independent ChIP-seq experiments. Significance levels were computed by Wilcoxon signed-rank test (***P < 0.001). (**C**) Schematic representation of time course experiments to monitor transcriptional responses of WT and *HDA1*-deleting cells undergoing starvation (−N) and nutrient refeeding (2nd +N). RNA samples were collected at the indicated time points. (**D**) Downregulation of *RPS19B* and *RPL16B* upon *HDA1* deletion in −N conditions. WT and *HDA1*-deleting cells were grown as in Fig. 5C, and mRNA levels were determined by RT-qPCR with three independent RNA samples. *SCR1* was used as an internal control. Error bars show standard deviation (S.D.) calculated from three biological replicates, each with three technical replicates. **P < 0.01, and ***P < 0.001 (two-tailed unpaired Student’s *t* tests). (**E**) *HDA1* deletion accelerates the recovery of *RPS19B* and *RPL16B* after nutrient refeeding. The indicated strains were grown as in Fig. 5C. mRNA levels were determined by RT-qPCR with three independent RNA samples, *SCR1* being used as an internal control. The ratios of transcript levels at 2nd +N (8 and 15 min) relative to the transcript levels in −N (4 h) were plotted. Error bars show standard deviation (S.D.) calculated from three biological replicates, each with three technical replicates. (**F**) Schematic representation of time course experiments to monitor transcriptional responses of WT and *HDA1*-deleting cells during carbon source shifts (Ra → Gal 2hr → Glu). Ra, Raffinose; Gal, Galactose; Glu, Glucose [12]. (**G**) Global downregulation of RP genes upon *HDA1* deletion in galactose medium. The box plot shows the log_2_ expression levels of the 137 RP genes in galactose medium (Gal 2 h) [12]. ***P < 0.001 (two-tailed unpaired Student’s *t* test). (**H**) Rapid reactivation kinetics of 137 RP genes in *HDA1*-deleting cells during the transition from galactose to glucose medium. The box plot represents the ratios of the transcript levels of the 137 RP genes in glucose medium (Glu 30 min, Glu 120 min) relative to their transcript levels in galactose medium (Gal 2 h) [12]. ***P < 0.001 (two-tailed unpaired Student’s *t* test).

We showed that Hda1C differentially deacetylates histone H3 and H4 at coding genes depending on their activity (Supplementary Fig. S1E and F). This suggests that Hda1C can change its substrate preference depending on local conditions. We thus assessed the substrate preference of Hda1C when it is targeted to the URS of RP genes by nutrient starvation. To this end, the histone acetylation patterns at the URS of the RP genes were analyzed. In +N, deletion of *HDA1* did not alter H4 acetylation at the RP gene URS, although it did slightly decrease H3 acetylation at these sites (Fig. 5A and B). This limited change likely reflects the fact that Hda1 generally does not bind to the URS in +N (Fig. 3B and C). By contrast, in −N, loss of Hda1 strongly increased H4 acetylation at the RP gene URS. H3 acetylation at these sites was also slightly increased (Fig. 5A and B). ChIP-qPCR analysis also showed these changes at individual RP gene URS (Supplementary Fig. S6A and B). Thus, Hda1C that has been relocalized to the URS of RP genes by starvation deacetylates both H4 and H3.

Although Hda1C associates strongly with the coding regions of RP genes, loss of this complex had no effect on RP gene expression in +N conditions [38]. However, when this complex is targeted to the URS in −N conditions, it may play two distinct roles. Hda1C may contribute to basal expression or mediate strong repression of RP genes in −N conditions. Alternatively, this complex may delay reactivation of RP genes when cells were shifted back to +N conditions. Thus, wild type and *hda1Δ* cells were grown in +N medium, transferred to −N medium for 4 h to repress RP gene transcription, and then shifted back to +N medium (2nd +N) to reactivate RP gene expression. RNA samples were collected at multiple time points (Fig. 5C) and the expression of two RP genes, *RPS19B* and *RPL16B*, was analyzed. In wild type cells, both genes were highly expressed in +N conditions, strongly downregulated in −N conditions, and rapidly reactivated when the cells were shifted back to 2nd +N medium (Supplementary Fig. S6C). *HDA1* deletion did not affect transcription of the two genes in +N conditions (Supplementary Fig. S6D), as expected [38]. Unexpectedly, however, in −N conditions, loss of Hda1 led to reduced expression of the two genes rather than increasing their expression (Fig. 5D), suggesting that Hda1C is important for basal expression of RP genes in −N condition. We also found that *HDA1* deletion accelerated the recovery of RP gene expression when the cells were returned to +N (Fig. 5E). To further explore the role of Hda1C at all 137 RP genes, we analyzed RNA-seq data that we generated previously from wild type and *hda1Δ* cells undergoing carbon source shifts leading to similar changes in RP gene expression [12] that we observed with +N/−N shifts. Briefly, cells were grown in synthetic complete medium containing raffinose, transferred to galactose for 2 h, and then transferred to glucose for 2 h (Fig. 5F). Similar to cells grown in +N/−N, deleting *HDA1* did not affect the expression of the 137 RP genes in raffinose medium (Supplementary Fig. S6E), but it further reduced the basal expression of these genes when the cells were incubated in galactose medium (Fig. 5G). *HDA1* deletion also accelerated the reactivation kinetics of the 137 RP genes (Fig. 5H). Thus, Hda1C may play two different regulatory roles in RP gene transcription, promoting basal expression under nutrient starvation conditions and delaying the kinetics of reactivation upon return to nutrition-rich conditions.

### Hda1C is required for optimal tRNA and rRNA expression

Hda1C is known to bind to RNA Pol III-dependent tRNA genes [31], although the function of Hda1C in tRNA transcription remains unknown. Our ChIP-seq results showed that Hda1C also binds to tRNA genes in a Pol II-independent manner in +N and −N (Fig. 2E). This finding prompted us to first compare the binding of Hda1 to the genes that are transcribed by the three eukaryotic RNA polymerases in +N. Specifically, we examined the binding of Hda1 to (i) the rDNA loci transcribed by RNA Pol I into rRNAs; (ii) the *YEF3* and *RPL16B* RP genes, which are transcribed by RNA Pol II into mRNAs; and (iii) the *tE(UUC)E3* and *tV(AAC)O* tRNA genes transcribed by RNA Pol III into tRNAs. We found that all regions displayed at least 1.5-fold stronger Hda1-myc signals compared to the untagged control (Supplementary Fig. S7A). Thus, Hda1C associates with genes that are transcribed by all three polymerases in +N.

**Figure 6.**
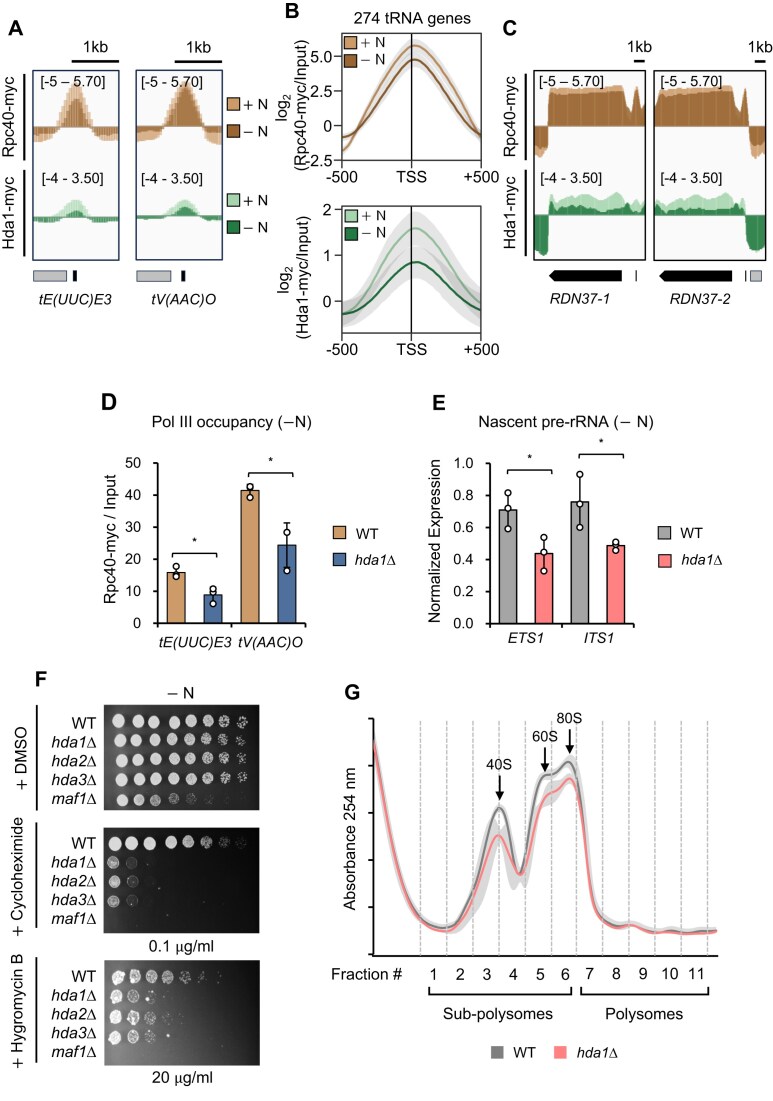
Hda1C regulates RNA Pol I and Pol III transcription and contributes to translation upon starvation. (**A–** **C**) Targeting of Hda1C to tRNAs and rDNA loci. (A) ChIP-seq tracks of the Rpc40-myc and Hda1-myc ChIP signals at *tE(UUC)E3* and *tV(AAC)O* in +N and −N. (B) Line plot showing the average Rpc40-myc and Hda1-myc ChIP-seq signals at the −500 to +500 bp region of the indicated tRNA genes in +N and −N. The standard deviation is indicated in gray. (C) ChIP-seq tracks of the Rpc40-myc and Hda1-myc ChIP signals at *RDN37-1* and *RDN37-2* in +N and −N. (**D**) Hda1C is required for optimal RNA Pol III occupancy to tRNA genes in −N. Crosslinked chromatin from WT and *HDA1*-deleting cells, expressing Rpc40-myc, was precipitated with anti-myc antibody. ChIP-qPCR analysis of the precipitated DNA was done on *tE(UUC)E3* and *tV(AAC)O* as in Fig. 4D. *P < 0.05 (two-tailed unpaired Student's *t* tests). (**E**) Downregulation of nascent pre-rRNA levels upon *HDA1* deletion in −N. Nascent pre-rRNA levels were determined by RT-qPCR with three independent RNA samples, *SCR1* being used as an internal control. *P < 0.05 (two-tailed unpaired Student’s *t* tests). (**F**) Mutants for Hda1C exhibit profound sensitivity to the translational inhibitors cycloheximide and hygromycin B in −N conditions. The indicated cells were spotted in 3-fold dilutions onto 0.15X YP (−N) plates containing DMSO, cycloheximide (0.1${\mathrm{\mu }}$g/ml), or hygromycin B (20${\mathrm{\mu }}$g /ml). The *maf1Δ* mutant was used as a positive control. (**G**) Loss of Hda1 slightly impairs ribosome assembly or translation in −N. Polysome profiles of wild type and *HDA1*-deleting cells were measured by tracing the UV absorbance at 254 nm (*A*_254_) after fractionating the whole cell extracts using a sucrose density gradient. The line plot depicts the average *A*_254_ signals for the indicated strains, with the standard deviation (S.D.) shown in gray. The average value and S.D. were calculated from three biological replicates.

Since we found that Hda1C binds to coding genes in a Pol II-associated manner (Fig. 1), we next asked whether Hda1C also binds to tRNA genes in a Pol III-associated manner and rDNA loci in a Pol I-associated manner. To test this, we examined the binding patterns of Hda1C and Rpc40 in wild type cells in +N and −N. Rpc40 is a subunit found in both RNA Pol I and Pol III. While RNA Pol III bound strongly to tRNA genes in +N conditions, this signal was decreased in −N conditions (Fig. 6A and B). Hda1 binding to tRNA genes showed a similar pattern (Fig. 6A and B). The same patterns were also observed at RNA Pol I-transcribed rDNAs (Fig. 6C), suggesting that RNA Pol III and Pol I transcription may respectively promote the binding of Hda1C to tRNA genes and rDNA loci in both +N and −N.

An important question is whether transcription of tRNA and rRNA genes is regulated by Hda1C. To determine this, we first examined whether Hda1C affects histone acetylation patterns and RNA Pol III occupancy at tRNA genes. Although Hda1C bound strongly to the tRNA genes, the loss of Hda1 did not increase histone acetylation patterns at tRNA genes in +N. However, in −N, mutants for Hda1 exhibited a slight increased levels of histone H3 and H4 acetylation (Supplementary Fig. S7B). Consistent with this, *HDA1* deletion did not change RNA Pol III binding to tRNA genes in +N conditions (Supplementary Fig. S7C). Unexpectedly, however, RNA Pol III occupancy at tRNA genes was decreased in mutants for Hda1 in −N (Fig. 6D). Next, we analyzed the nascent rRNA levels in wild type and *hda1Δ* cells in +N and −N. The expression of *ETS1* and *ITS1*, which are sequences in nascent 35s rRNA, was measured to quantify nascent rRNA levels, as described previously [39, 40]. In +N, *HDA1* deletion had no effect on rRNA levels but it significantly reduced nascent rRNA levels in −N (Fig. 6E and Supplementary Fig. S7D). Thus, Hda1C is important for optimal expression of tRNAs and rRNAs in starvation conditions. Further studies on the underlying mechanism(s) are needed.

### Hda1C is involved in translation

We observed above that Hda1C promotes basal expression of RP genes in −N (Fig. 5), delays recovery of these genes when cells are returned to +N (Fig. 5) and is needed for optimal tRNA and rRNA synthesis in −N (Fig. 6D and E). Since RPs, tRNAs, and rRNAs are all crucial elements of translation, these observations suggest that Hda1C could shape translation. This notion led us to investigate phenotypes of mutants for Hda1C upon treatment with chemicals affecting either transcription or translation. 6-Azauracil (6-AU) and mycophenolic acid (MPA) are known to likely inhibit transcription elongation by reducing NTP pools. Given Hda1C’s role in deacetylating histone H4 at actively transcribed genes and the slight suppression of growth defects in mutants for the positive elongation factor Bur1, it suggests a potential involvement in transcription elongation [12]. However, 6-AU and MPA did not alter the growth of mutants for Hda1C relative to the wild type cells (Supplementary Fig. S8A).

By contrast, treatment of cycloheximide and hygromycin B, which inhibit translation [41–43], resulted in obvious phenotypes. In +N conditions, Hda1C mutants showed a slight sensitivity to high concentrations of cycloheximide and hygromycin B compared to wild type cells (Supplementary Fig. S8B and C). Strikingly, in −N conditions, Hda1C mutants exhibited severe growth defects with these chemicals (Fig. 6F, and Supplementary Fig. S8B and C). As expected, deleting *MAF1*, which negatively regulates RNA Pol III in yeast, greatly increased sensitivity to cycloheximide under −N conditions [44]. For comparison, we also examined the effect of cycloheximide on cells that lack components of other HDACs that likely affect RP gene expression, Rpd3, Sir2, Hos2, and Hst1. While Sir2 mutants did not show obvious phenotypes with cycloheximide treatment in both +N and −N conditions, *RPD3* deleting cells were sensitive to cycloheximide (Supplementary Fig. S8D). This likely reflects the inhibitory role of Rpd3 in RP gene transcription during nutrient starvation or quiescence [45–49]. The loss of Hos2 or Hst1, which are subunits of the Set3 HDAC, also increased sensitivity to cycloheximide (Supplementary Fig. S8D). However, deletions in Hda1C have more severe effects than mutants for other HDACs in −N conditions.

To further determine the effect of Hda1C on translation, we next performed polysome profiling by sucrose gradient ultracentrifugation. In the wild type cells, starvation significantly decreased the polysome fraction and increased the sub-polysome fraction, which indicates global inhibition of translation (Supplementary Fig. S8E). *HDA1* deletion had no effect on the polysome/sub-polysome profile in +N conditions (Supplementary Fig. S8F). Interestingly, in −N conditions, the sub-polysome fraction was reduced in *hda1Δ* cells compared to wild type cells (Fig. 6G). These findings suggest that Hda1C is required for efficient assembly of ribosome and translation by controlling transcription of translation-related genes under nutrient starvation conditions.

## Discussion

**Figure 7.**
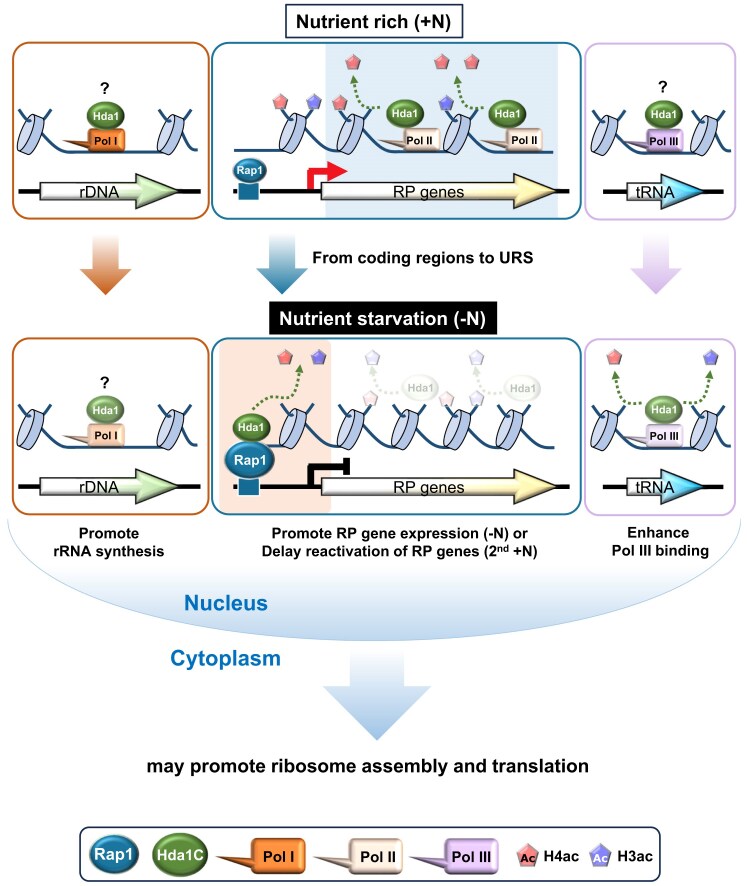
Models of Hda1C-mediated coordination of transcription and translation. Hda1C interacts with genes that are transcribed by all three RNA polymerases (RNA Pol I, II, and III). In nutrient-rich conditions, Hda1C primarily associates with elongating RNA Pol II and deacetylates histone H4 at the coding regions of RP genes. Hda1C also localizes to rDNA and tRNA loci, transcribed by RNA Pol I and Pol III, respectively. However, its precise role in regulating transcription of these genes under nutrient rich conditions remains to be clarified. Upon nutrient starvation, Hda1C relocates to the URS of RP genes by interacting with Rap1, where it deacetylates both histone H3 and H4. This deacetylation either enhances basal transcription or delays reactivation of RP genes when the cells are shifted back to nutrient-rich conditions. Hda1C remains associated with rDNA and tRNA loci and promotes their transcription by RNA Pol I and Pol III, respectively. All of these activities of Hda1C occur within the nucleus. The fact that Hda1C affects RP gene expression and rRNA synthesis suggests that Hda1C shapes ribosome assembly. Moreover, by facilitating tRNA transcription, Hda1C may also directly support translation. These observations propose that Hda1C functions as a key integrator that coordinates transcriptional regulation and ribosome activity, particularly during nutrient starvation, to ensure cellular adaptation to changing environmental conditions.

Histone acetylation and deacetylation play crucial roles in regulating RNA Pol II transcription, with their effects varying depending on their location within a gene. At promoters, these opposing processes fine-tune transcription initiation by either promoting or inhibiting the formation of the preinitiation complex. In the gene body, histone acetylation facilitates nucleosome eviction to promote RNA Pol II elongation, whereas deacetylation helps restore repressive chromatin structures after the passage of RNA Pol II [2, 3]. Histone acetylation and deacetylation are carried out by distinct HATs and HDACs, which are likely targeted to specific regions to perform these functions [5]. Our results, however, demonstrate that Hda1C exhibits dynamic localization depending on transcriptional activity, particularly at RP genes. Under normal conditions, Hda1C is recruited to the coding regions of active RP genes. In contrast, upon nutrient starvation, Hda1C relocates to the URS of inactive RP genes, likely to enhance basal expression or to delay the reactivation of these genes (Fig. 7). In addition, Hda1C also binds to RNA Pol III-transcribed tRNA genes and RNA Pol I-dependent rDNA, contributing to the maintenance of optimal expression. Hda1C is also required for normal growth in the presence of translation inhibitors, and its loss leads to altered ribosome profile patterns. These findings suggest that Hda1C plays a pivotal role in coordinating transcription and translation in response to nutrient availability by modulating the activity of RNA Pol I, II, and III (Fig. 7).

Interestingly, Hda1C is targeted to two distinct regions of RP genes depending on transcription levels. It strongly associates with the coding regions of actively transcribed RP genes in +N conditions, likely through interactions with elongating RNA Pol II [12, 13]. Upon nutrient starvation, leading to inactivation of RP genes, Hda1C primarily localizes to the URS of these genes (Fig. 3B and C). This relocalization is likely mediated by interaction with Rap1, as the Hda1C-binding regions of RP genes in −N conditions show a strong enrichment of Rap1-binding motifs (Fig. 4A and B). Moreover, Hda1C co-immunoprecipitates with Rap1 and depletion of Rap1 leads to the dissociation of Hda1C from the URS of RP genes (Fig. 4E and F, and Supplementary Fig. S5). Rap1 is a sequence-specific DNA binding protein that performs distinct regulatory functions at different genomic loci. At telomeric and mating type loci, where Rap1 acts as a repressor, it recruits the Sir complex through direct interaction with Sir3 and Sir4 to facilitate transcriptional silencing [50, 51]. By contrast, at the URS of actively transcribed RP genes, Rap1 contributes to the recruitment of the general transcription factor TFIID (Transcription Factor II D) and other transcription activators, including Ifh1 and Fhl1, to promote RP gene transcription [52–55]. Whether Rap1 also functions as a repressor at mRNA genes or how it performs this function remains unknown. Our findings show that Rap1 remains associated with the URS of RP genes and promotes the recruitment of Hda1C upon nutrient starvation (Fig. 4).

An important question is what triggers the interaction between Hda1C and Rap1 upon starvation and what leads to the dissociation of these two proteins when RP genes are reactivated. One possibility is that Hda1C competes with transcriptional activators for binding to Rap1. Alternatively, post-translational modifications of Hda1C such as SUMOylation, phosphorylation, and acetylation may play a crucial role in regulating this interaction [56–58]. Among these modifications, SUMOylation is particularly interesting because it can influence protein–protein interactions by either disrupting existing interactions or promoting new ones through SUMO-interaction motifs (SIMs) [59]. Importantly, Hda1 contains multiple SUMOylation sites and SIMs, while Hda2 also includes a SUMOylation site [60, 61]. Furthermore, factors that influence the binding of Hda1C to the genome, including RNA Pol II subunits and Rap1, also have SUMOylation sites [60–63]. Further investigation will be crucial to elucidate the precise mechanisms by which Hda1C is relocalized in response to environmental cues.

Several chromatin factors can be targeted to both promoters and coding regions of genes. Although Rpd3 also binds to both upstream and coding regions of target genes, it is likely that two distinct Rpd3-containing HDACs, Rpd3 large (Rpd3L) and Rpd3 small (Rpd3S), function at different regions of genes. Upon quiescence entry, Rpd3 is targeted to promoters, ∼150 bp upstream of TSS, of many genes, including RP genes to mediate global transcriptional shut off [48]. Rpd3L is mainly targeted to promoter regions via interaction with gene-specific repressors and H3K4 methylation enriched at promoter regions [64]. In contrast, Rpd3S is preferentially targeted to coding regions via elongating RNA Pol II and H3K36 methylation peaking at coding regions [65–68]. These suggest that two different Rpd3-containing HDACs play distinct roles at promoters and coding regions. However, our findings indicate that Hda1C itself is targeted to two distinct regions of the same gene depending on transcription frequency. The RSC chromatin remodeling complex also relocates from coding regions to promoters upon heat shock, although the mechanism underlying this relocalization remains unclear [69]. Additionally, transcriptional outcomes of such relocation events require further elucidation.

An additional significant finding from this study is that, in addition to its dynamic relocalization between the coding regions and the URS of RP genes, Hda1C also associates with rDNA and tRNA loci, which are linked to RNA Pol I and RNA Pol III activity, respectively. Notably, loss of Hda1C results in a modest but significant reduction in rRNA synthesis by RNA Pol I and a decrease in RNA Pol III binding to tRNA genes (Fig. 6). These findings suggest that Hda1C has a positive regulatory role at these loci, similar to its function at RP genes under nutrient starvation conditions. While mutants for Hda1C exhibit increased acetylation levels at both RP and tRNA genes, the resulting transcriptional outcomes are contrasting. Although histone acetylation is generally associated with active transcription, elevated acetylation levels at regulatory regions, including the URS of RP genes, may also facilitate the binding of transcriptional repressors, contributing to gene repression. Overall, our findings highlight the broader influence of Hda1C on transcription across all three RNA polymerases, potentially impacting translation by regulating RP gene expression and tRNA and rRNA synthesis.

Although Hda1C primarily functions within the nucleus to regulate transcription, mutants for Hda1C exhibit pronounced sensitivity to translation inhibitors and show dysregulation of translation by ribosomes under nutrient starvation conditions (Fig. 6). These findings imply that Hda1C may shape translation by altering the transcription of translation machinery components. However, we cannot exclude the possibility that Hda1C also influences translation by regulating nonhistone proteins involved in translation. It should be noted that while deletion mutations of other HDACs also increased sensitivity to translation inhibitors, Hda1C deletion mutations had a much stronger effect (Supplementary Fig. S8D). Thus, Hda1C may play a more extensive role in cellular regulation than was previously understood. Notably, studies have shown that other transcription factors, including the Rpb4/7 subunit of RNA Pol II and Ctk1 in yeast and CDK9, p53, and TAF7 in higher eukaryotes, also control translation. However, the mechanism involves the relocation of these molecules from the nucleus to the cytoplasm. Unlike these factors, Hda1C predominantly remains within the nucleus [70]. This raises the possibility that Hda1C could serve as a novel link between transcription and translation without requiring physical movement to the cytoplasm. A recent study showed that Hos2, a catalytic subunit of Set3 HDAC, modulates protein expression noise by promoting expression of RP genes [71]. The fact that Hda1C regulates transcription mediated by RNA Pol I, RNA Pol II, and RNA Pol III, thereby influencing ribosomal function, positions it as a unique factor that coordinates translation from within the nucleus. In conclusion, Hda1C may act as a key coordinator that balances transcription and translation in the cell. Further research is necessary to fully understand the mechanisms by which Hda1C exerts this regulatory control and to explore its broader impact on cellular processes.

## Supplementary Material

gkaf256_Supplemental_File

## Data Availability

Sequencing data have been deposited in GEO (https://www.ncbi.nlm.nih.gov/geo/) under accession number GSE276792.

## References

[1] Kim U, Lee DS Epigenetic regulations in mammalian cells: roles and profiling techniques. Mol Cells. 2023; 46:86–98. 10.14348/molcells.2023.0013. 36859473 PMC9982057

[2] Kouzarides T Chromatin modifications and their function. Cell. 2007; 128:693–705. 10.1016/j.cell.2007.02.005. 17320507

[3] Li B, Carey M, Workman JL The role of chromatin during transcription. Cell. 2007; 128:707–19. 10.1016/j.cell.2007.01.015. 17320508

[4] Buratowski S, Kim T The role of cotranscriptional histone methylations. Cold Spring Harbor Symp Quant Biol. 2010; 75:95–102. 10.1101/sqb.2010.75.036. 21447819 PMC3229092

[5] Woo H, Ha D, L S et al. Modulation of gene expression dynamics by co-transcriptional histone methylations. Exp Mol Med. 2017; 49:e326 10.1038/emm.2017.19. 28450734 PMC6130219

[6] Rundlett SE, Carmen AA, Kobayashi R et al. HDA1 and RPD3 are members of distinct yeast histone deacetylase complexes that regulate silencing and transcription. Proc Natl Acad Sci USA. 1996; 93:14503–8. 10.1073/pnas.93.25.14503. 8962081 PMC26162

[7] Wu J, Carmen AA, Kobayashi R et al. HDA2 and HDA3 are related proteins that interact with and are essential for the activity of the yeast histone deacetylase HDA1. Proc Natl Acad Sci USA. 2001; 98:4391–6. 10.1073/pnas.081560698. 11287668 PMC31845

[8] Carmen AA, Rundlett SE, Grunstein M HDA1 and HDA3 are components of a yeast histone deacetylase (HDA) complex. J Biol Chem. 1996; 271:15837–44. 10.1074/jbc.271.26.15837. 8663039

[9] Lee JH, Bollschweiler D, Schafer T et al. Structural basis for the regulation of nucleosome recognition and HDAC activity by histone deacetylase assemblies. Sci Adv. 2021; 7:eabd4413 10.1126/sciadv.abd4413. 33523989 PMC7793584

[10] Lee JH, Maskos K, Huber R Structural and functional studies of the yeast class II Hda1 histone deacetylase complex. J Mol Biol. 2009; 391:744–57. 10.1016/j.jmb.2009.06.059. 19573535

[11] Shen H, Zhu Y, Wang C et al. Structural and histone binding ability characterization of the ARB2 domain of a histone deacetylase Hda1 from *Saccharomyces cerevisiae*. Sci Rep. 2016; 6:33905 10.1038/srep33905. 27665728 PMC5036196

[12] Ha SD, Ham S, Kim MY et al. Transcription-dependent targeting of Hda1C to hyperactive genes mediates H4-specific deacetylation in yeast. Nat Commun. 2019; 10:4270 10.1038/s41467-019-12077-w. 31537788 PMC6753149

[13] Lee MK, Kim T Histone H4-specific deacetylation at active coding regions by Hda1C. Mol Cells. 2020; 43:841–7. 32913143 10.14348/molcells.2020.0141 PMC7604025

[14] Wu J, Suka N, Carlson M et al. TUP1 utilizes histone H3/H2B-specific HDA1 deacetylase to repress gene activity in yeast. Mol Cell. 2001; 7:117–26. 10.1016/S1097-2765(01)00160-5. 11172717

[15] Harel-Sharvit L, Eldad N, Haimovich G et al. RNA polymerase II subunits link transcription and mRNA decay to translation. Cell. 2010; 143:552–63. 10.1016/j.cell.2010.10.033. 21074047

[16] Buratowski S Progression through the RNA polymerase II CTD cycle. Mol Cell. 2009; 36:541–6. 10.1016/j.molcel.2009.10.019. 19941815 PMC3232742

[17] Röther S, Strässer K The RNA polymerase II CTD kinase Ctk1 functions in translation elongation. Genes Dev. 2007; 21:1409–21. 10.1101/gad.428407. 17545469 PMC1877752

[18] Marcel V, Catez F, Diaz JJ p53, a translational regulator: contribution to its tumour-suppressor activity. Oncogene. 2015; 34:5513–23. 10.1038/onc.2015.25. 25728674

[19] Cheng D, Semmens K, McManus E et al. The nuclear transcription factor, TAF7, is a cytoplasmic regulator of protein synthesis. Sci Adv. 2021; 7:eabi5751 10.1126/sciadv.abi5751. 34890234 PMC8664259

[20] Booher KR, Kaiser P A PCR-based strategy to generate yeast strains expressing endogenous levels of amino-terminal epitope-tagged proteins. Biotechnol J. 2008; 3:524–9. 10.1002/biot.200800012. 18320568

[21] Bong D, Sohn J, Lee SJ Brief guide to RT-qPCR. Mol Cells. 2024; 47:100141 10.1016/j.mocell.2024.100141. 39476972 PMC11612376

[22] Kim T, Buratowski S Dimethylation of H3K4 by Set1 recruits the Set3 histone deacetylase complex to 5′ transcribed regions. Cell. 2009; 137:259–72. 10.1016/j.cell.2009.02.045. 19379692 PMC2802783

[23] Bailey TL, Machanick P Inferring direct DNA binding from ChIP-seq. Nucleic Acids Res. 2012; 40:e128–10.1093/nar/gks433. 22610855 PMC3458523

[24] Langmead B, Salzberg SL Fast gapped-read alignment with Bowtie 2. Nat Methods. 2012; 9:357–9. 10.1038/nmeth.1923. 22388286 PMC3322381

[25] Danecek P, Bonfield JK, Liddle J et al. Twelve years of SAMtools and BCFtools. Gigascience. 2021; 10:giab008 10.1093/gigascience/giab008. 33590861 PMC7931819

[26] Zhang Y, Liu T, Meyer CA et al. Model-based analysis of ChIP-Seq (MACS). Genome Biol. 2008; 9:R137 10.1186/gb-2008-9-9-r137. 18798982 PMC2592715

[27] Ramirez F, Ryan DP, Gruning B et al. deepTools2: a next generation web server for deep-sequencing data analysis. Nucleic Acids Res. 2016; 44:W160–5. 10.1093/nar/gkw257. 27079975 PMC4987876

[28] Love MI, Huber W, Anders S Moderated estimation of fold change and dispersion for RNA-seq data with DESeq2. Genome Biol. 2014; 15:550 10.1186/s13059-014-0550-8. 25516281 PMC4302049

[29] Kuleshov MV, Jones MR, Rouillard AD et al. Enrichr: a comprehensive gene set enrichment analysis web server 2016 update. Nucleic Acids Res. 2016; 44:W90–7. 10.1093/nar/gkw377. 27141961 PMC4987924

[30] McLeay RC, Bailey TL Motif Enrichment Analysis: a unified framework and an evaluation on ChIP data. BMC Bioinf. 2010; 11:165 10.1186/1471-2105-11-165. PMC2868005 20356413

[31] Venters BJ, Wachi S, Mavrich TN et al. A comprehensive genomic binding map of gene and chromatin regulatory proteins in Saccharomyces. Mol Cell. 2011; 41:480–92. 10.1016/j.molcel.2011.01.015. 21329885 PMC3057419

[32] Lempiainen H, Shore D Growth control and ribosome biogenesis. Curr Opin Cell Biol. 2009; 21:855–63. 10.1016/j.ceb.2009.09.002. 19796927

[33] Warner JR The economics of ribosome biosynthesis in yeast. Trends Biochem Sci. 1999; 24:437–40. 10.1016/S0968-0004(99)01460-7. 10542411

[34] Knight B, Kubik S, Ghosh B et al. Two distinct promoter architectures centered on dynamic nucleosomes control ribosomal protein gene transcription. Genes Dev. 2014; 28:1695–709. 10.1101/gad.244434.114. 25085421 PMC4117944

[35] Lieb JD, Liu X, Botstein D et al. Promoter-specific binding of Rap1 revealed by genome-wide maps of protein-DNA association. Nat Genet. 2001; 28:327–34. 10.1038/ng569. 11455386

[36] Wade JT, Hall DB, Struhl K The transcription factor Ifh1 is a key regulator of yeast ribosomal protein genes. Nature. 2004; 432:1054–8. 10.1038/nature03175. 15616568

[37] Morawska M, Ulrich HD An expanded tool kit for the auxin-inducible degron system in budding yeast. Yeast. 2013; 30:341–51. 10.1002/yea.2967. 23836714 PMC4171812

[38] Lenstra TL, Benschop JJ, Kim T et al. The specificity and topology of chromatin interaction pathways in yeast. Mol Cell. 2011; 42:536–49. 10.1016/j.molcel.2011.03.026. 21596317 PMC4435841

[39] Laribee RN, Hosni-Ahmed A, Workman JJ et al. Ccr4-not regulates RNA polymerase I transcription and couples nutrient signaling to the control of ribosomal RNA biogenesis. PLoS Genet. 2015; 11:e1005113 10.1371/journal.pgen.1005113. 25815716 PMC4376722

[40] Shu WJ, Chen R, Yin ZH et al. Rph1 coordinates transcription of ribosomal protein genes and ribosomal RNAs to control cell growth under nutrient stress conditions. Nucleic Acids Res. 2020; 48:8360–73. 10.1093/nar/gkaa558. 32619236 PMC7470948

[41] Bolger TA, Folkmann AW, Tran EJ et al. The mRNA export factor Gle1 and inositol hexakisphosphate regulate distinct stages of translation. Cell. 2008; 134:624–33. 10.1016/j.cell.2008.06.027. 18724935 PMC2601711

[42] Fujii K, Susanto TT, Saurabh S et al. Decoding the function of expansion segments in ribosomes. Mol Cell. 2018; 72:1013–20. 10.1016/j.molcel.2018.11.023. 30576652 PMC6407129

[43] Gonzalez A, Jimenez A, Vazquez D et al. Studies on the mode of action of hygromycin B, an inhibitor of translocation in eukaryotes. Biochim Biophys Acta. 1978; 521:459–69. 10.1016/0005-2787(78)90287-3. 367435

[44] Upadhya R, Lee JH, Willis IM Maf1 is an essential mediator of diverse signals that repress RNA polymerase III transcription. Mol Cell. 2002; 10:1489–94. 10.1016/S1097-2765(02)00787-6. 12504022

[45] Kurdistani SK, Robyr D, Tavazoie S et al. Genome-wide binding map of the histone deacetylase Rpd3 in yeast. Nat Genet. 2002; 31:248–54. 10.1038/ng907. 12089521

[46] Tsang CK, Bertram PG, Ai W et al. Chromatin-mediated regulation of nucleolar structure and RNA pol I localization by TOR. EMBO J. 2003; 22:6045–56. 10.1093/emboj/cdg578. 14609951 PMC275436

[47] Humphrey EL, Shamji AF, Bernstein BE et al. Rpd3p relocation mediates a transcriptional response to rapamycin in yeast. Chem Biol. 2004; 11:295–9. 10.1016/j.chembiol.2004.03.001. 15123258

[48] McKnight JN, Boerma JW, Breeden LL et al. Global promoter targeting of a conserved lysine deacetylase for transcriptional shutoff during quiescence entry. Mol Cell. 2015; 59:732–43. 10.1016/j.molcel.2015.07.014. 26300265 PMC4560983

[49] Huber A, French SL, Tekotte H et al. Sch9 regulates ribosome biogenesis via Stb3, Dot6 and Tod6 and the histone deacetylase complex RPD3L. EMBO J. 2011; 30:3052–64. 10.1038/emboj.2011.221. 21730963 PMC3160192

[50] Moretti P, Shore D Multiple interactions in sir protein recruitment by Rap1p at silencers and telomeres in yeast. Mol Cell Biol. 2001; 21:8082–94. 10.1128/MCB.21.23.8082-8094.2001. 11689698 PMC99974

[51] Luo K, Vega-Palas MA, Grunstein M Rap1–Sir4 binding independent of other Sir, yKu, or histone interactions initiates the assembly of telomeric heterochromatin in yeast. Genes Dev. 2002; 16:1528–39. 10.1101/gad.988802. 12080091 PMC186350

[52] Mencia M, Moqtaderi Z, Geisberg JV et al. Activator-specific recruitment of TFIID and regulation of ribosomal protein genes in yeast. Mol Cell. 2002; 9:823–33. 10.1016/S1097-2765(02)00490-2. 11983173

[53] Martin DE, Soulard A, Hall MN TOR regulates ribosomal protein gene expression via PKA and the Forkhead transcription factor FHL1. Cell. 2004; 119:969–79. 10.1016/j.cell.2004.11.047. 15620355

[54] Schawalder SB, Kabani M, Howald I et al. Growth-regulated recruitment of the essential yeast ribosomal protein gene activator Ifh1. Nature. 2004; 432:1058–61. 10.1038/nature03200. 15616569

[55] Garbett KA, Tripathi MK, Cencki B et al. Yeast TFIID serves as a coactivator for Rap1p by direct protein–protein interaction. Mol Cell Biol. 2007; 27:297–311. 10.1128/MCB.01558-06. 17074814 PMC1800639

[56] Henriksen P, Wagner SA, Weinert BT et al. Proteome-wide analysis of lysine acetylation suggests its broad regulatory scope in *Saccharomyces cerevisiae*. Mol Cell Proteomics. 2012; 11:1510–22. 22865919 10.1074/mcp.M112.017251 PMC3494197

[57] Bhagwat NR, Owens SN, Ito M et al. SUMO is a pervasive regulator of meiosis. eLife. 2021; 10:e57720 10.7554/eLife.57720. 33502312 PMC7924959

[58] Lanz MC, Yugandhar K, Gupta S et al. In-depth and 3-dimensional exploration of the budding yeast phosphoproteome. EMBO Rep. 2021; 22:e51121 10.15252/embr.202051121. 33491328 PMC7857435

[59] Jentsch S, Psakhye I Control of nuclear activities by substrate-selective and protein-group SUMOylation. Annu Rev Genet. 2013; 47:167–86. 10.1146/annurev-genet-111212-133453. 24016193

[60] Zhao Q, Xie YB, Zheng YY et al. GPS-SUMO: a tool for the prediction of sumoylation sites and SUMO-interaction motifs. Nucleic Acids Res. 2014; 42:W325–30. 10.1093/nar/gku383. 24880689 PMC4086084

[61] Zhou W, Ryan JJ, Zhou H Global analyses of sumoylated proteins in *Saccharomyces cerevisiae*. Induction of protein sumoylation by cellular stresses. J Biol Chem. 2004; 279:32262–8. 10.1074/jbc.M404173200. 15166219 PMC2810850

[62] Lewicki MC, Srikumar T, Johnson E et al. The *S. cerevisiae* SUMO stress response is a conjugation-deconjugation cycle that targets the transcription machinery. J Proteomics. 2015; 118:39–48. 10.1016/j.jprot.2014.11.012. 25434491

[63] Denison C, Rudner AD, Gerber SA et al. A proteomic strategy for gaining insights into protein sumoylation in yeast. Mol Cell Proteomics. 2005; 4:246–54. 10.1074/mcp.M400154-MCP200. 15542864

[64] Lee BB, Choi A, Kim JH et al. Rpd3L HDAC links H3K4me3 to transcriptional repression memory. Nucleic Acids Res. 2018; 46:8261–74. 10.1093/nar/gky573. 29982589 PMC6144869

[65] Carrozza MJ, Li B, Florens L et al. Histone H3 methylation by Set2 directs deacetylation of coding regions by Rpd3S to suppress spurious intragenic transcription. Cell. 2005; 123:581–92. 10.1016/j.cell.2005.10.023. 16286007

[66] Keogh MC, Kurdistani SK, Morris SA et al. Cotranscriptional set2 methylation of histone H3 lysine 36 recruits a repressive Rpd3 complex. Cell. 2005; 123:593–605. 10.1016/j.cell.2005.10.025. 16286008

[67] Li B, Gogol M, Carey M et al. Combined action of PHD and chromo domains directs the Rpd3S HDAC to transcribed chromatin. Science. 2007; 316:1050–4. 10.1126/science.1139004. 17510366

[68] Drouin S, Laramée L, Jacques PÉ et al. DSIF and RNA polymerase II CTD phosphorylation coordinate the recruitment of Rpd3S to actively transcribed genes. PLoS Genet. 2010; 6:e1001173 10.1371/journal.pgen.1001173. 21060864 PMC2965751

[69] Vinayachandran V, Reja R, Rossi MJ et al. Widespread and precise reprogramming of yeast protein–genome interactions in response to heat shock. Genome Res. 2018; 28:357–66. 10.1101/gr.226761.117. 29444801 PMC5848614

[70] Chong YT, Koh JL, Friesen H et al. Yeast proteome dynamics from single cell imaging and automated analysis. Cell. 2015; 161:1413–24. 10.1016/j.cell.2015.04.051. 26046442

[71] Lin WH, Opoc FJG, Liao CW et al. Histone deacetylase Hos2 regulates protein expression noise by potentially modulating the protein translation machinery. Nucleic Acids Res. 2024; 52:7556–71. 10.1093/nar/gkae432. 38783136 PMC11260488

