# Supplementary material for: Nutrient starvation-induced Hda1C rewiring: coordinated regulation of transcription and translation
Source: Nucleic Acids Res. 2025 Apr 18;53(7):gkaf256. doi: 10.1093/nar/gkaf256 (PMC12006795; doi:10.1093/nar/gkaf256)
Supplement: gkaf256_Supplemental_File [file gkaf256_supplemental_file.pdf]

Supplementary Figure S1

A

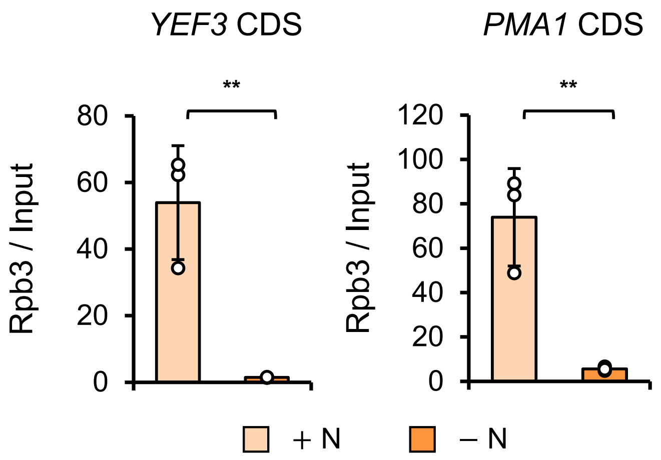

B

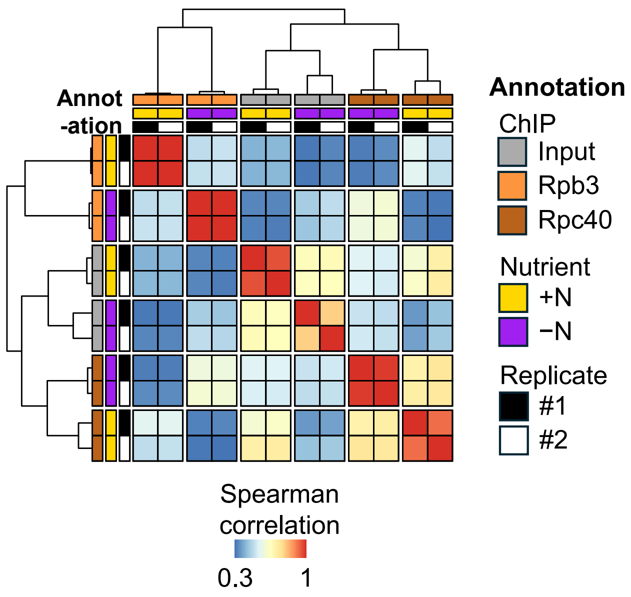

C

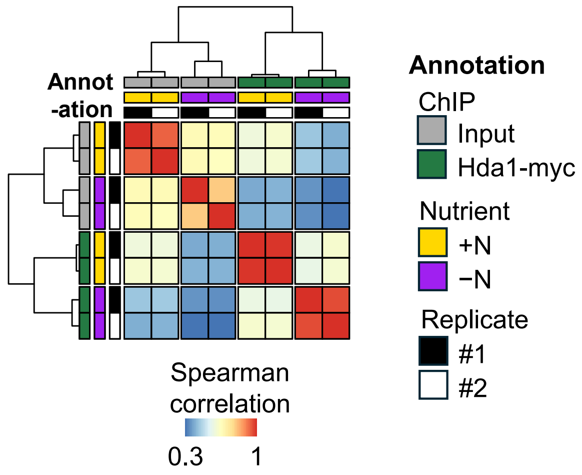

D

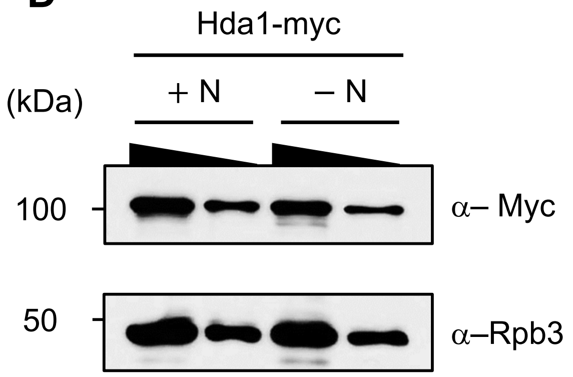

Supplementary Figure S1

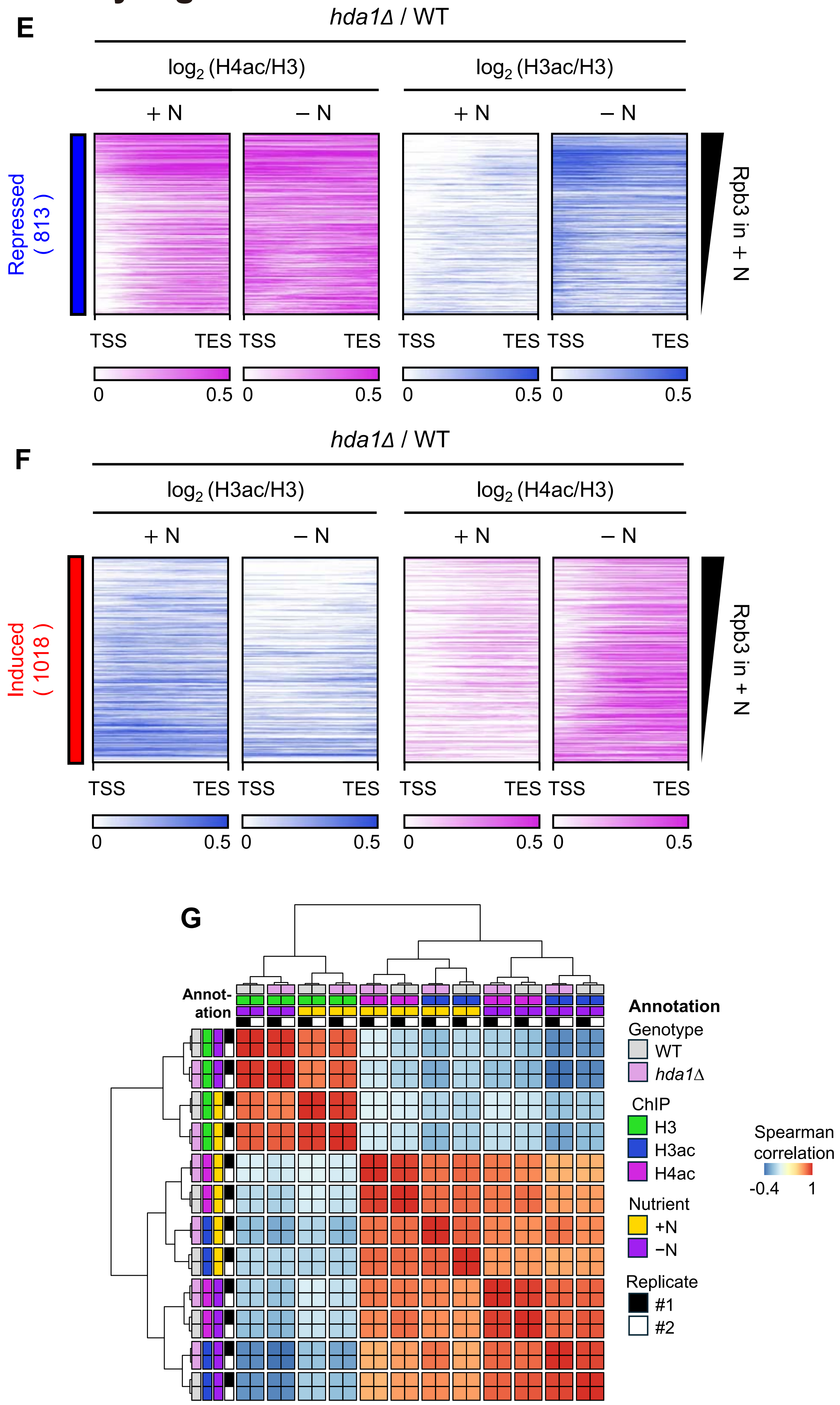

Supplementary Figure S2

A

		Pol II-associated	Pol II-independent
Bins (200bp)	+ N	59100	**1233**
	– N	58058	**2275**
Hda1 Peaks	+ N	751	**477**
	– N	859	**934**

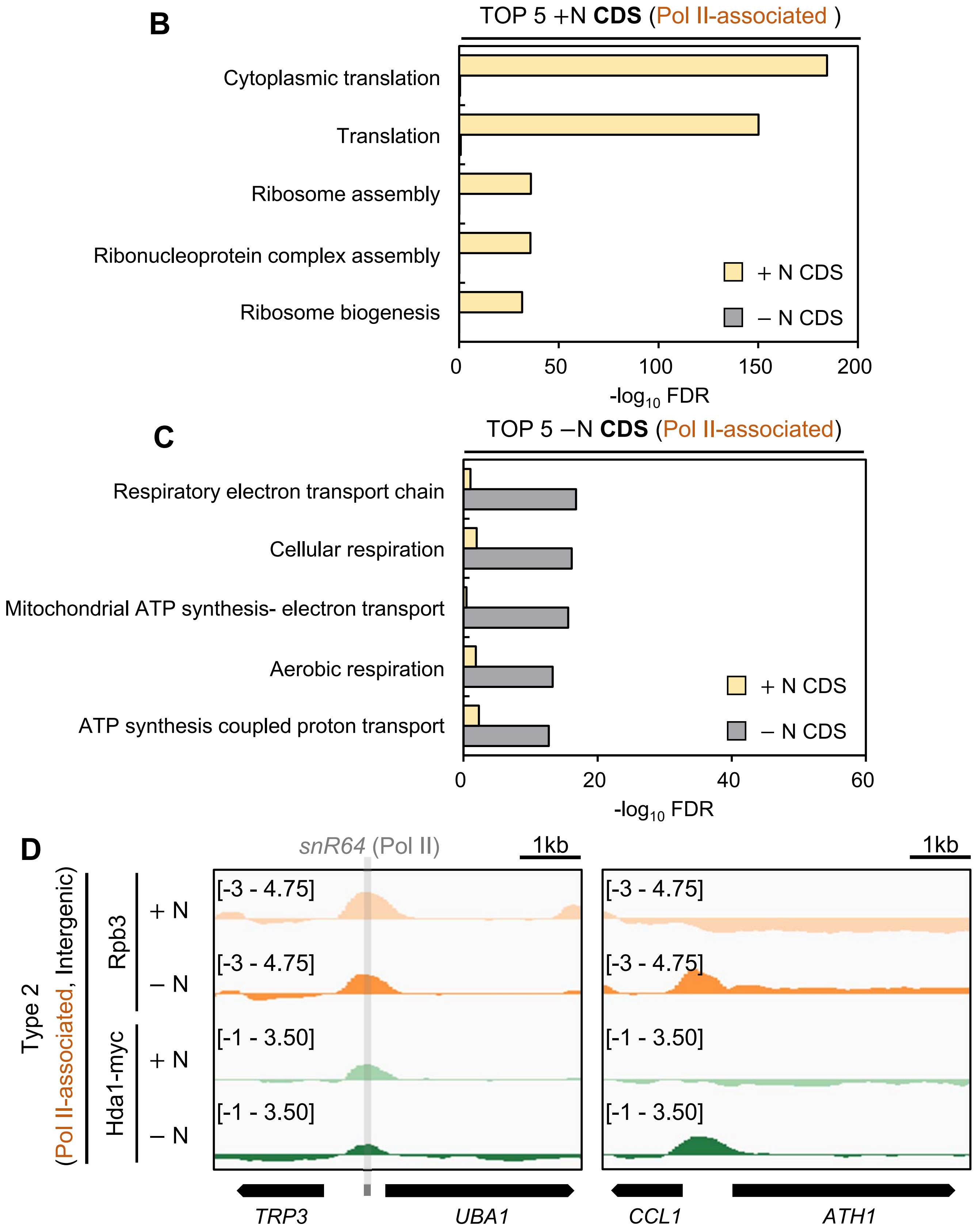

Supplementary Figure S3

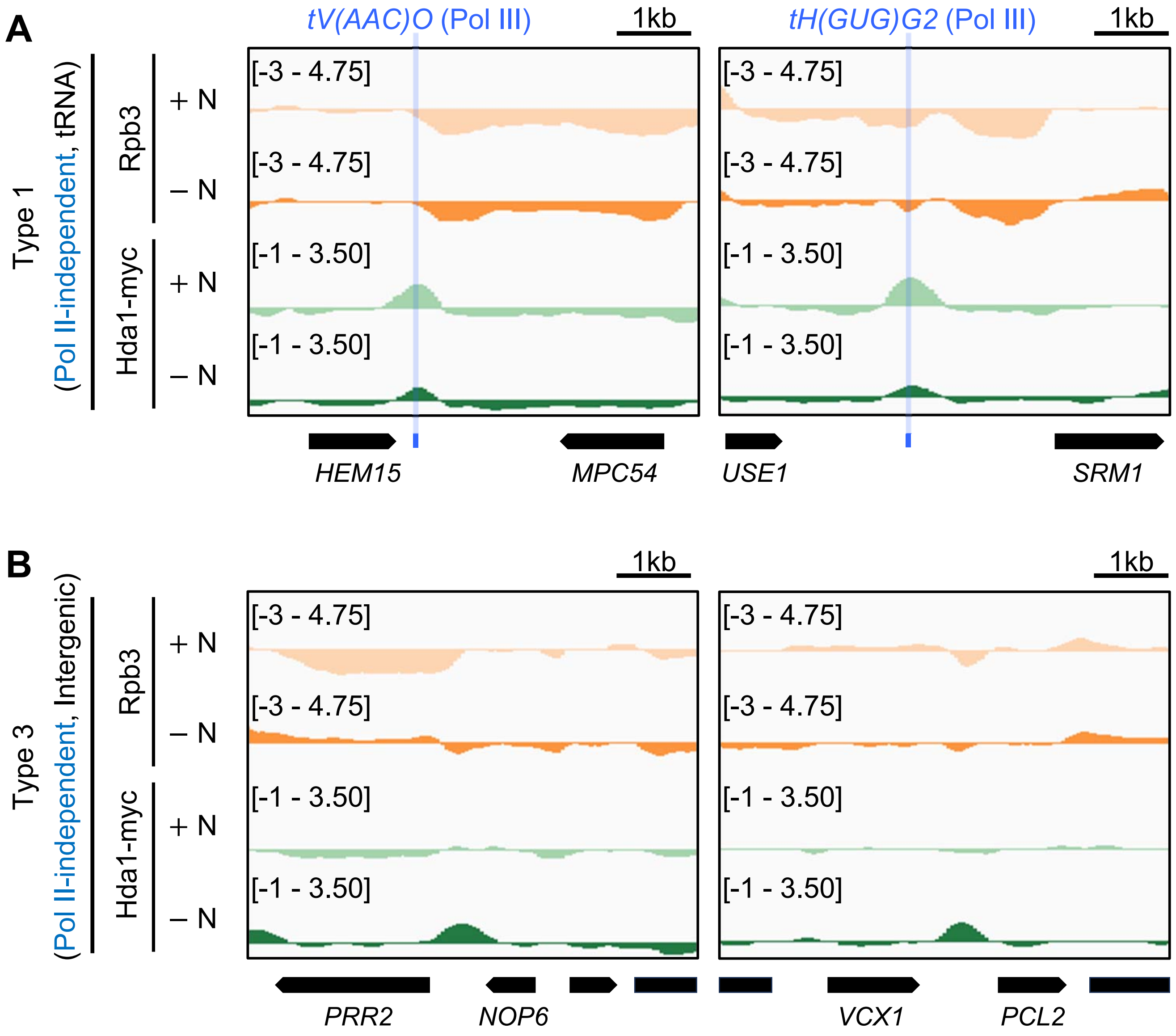

Supplementary Figure S4

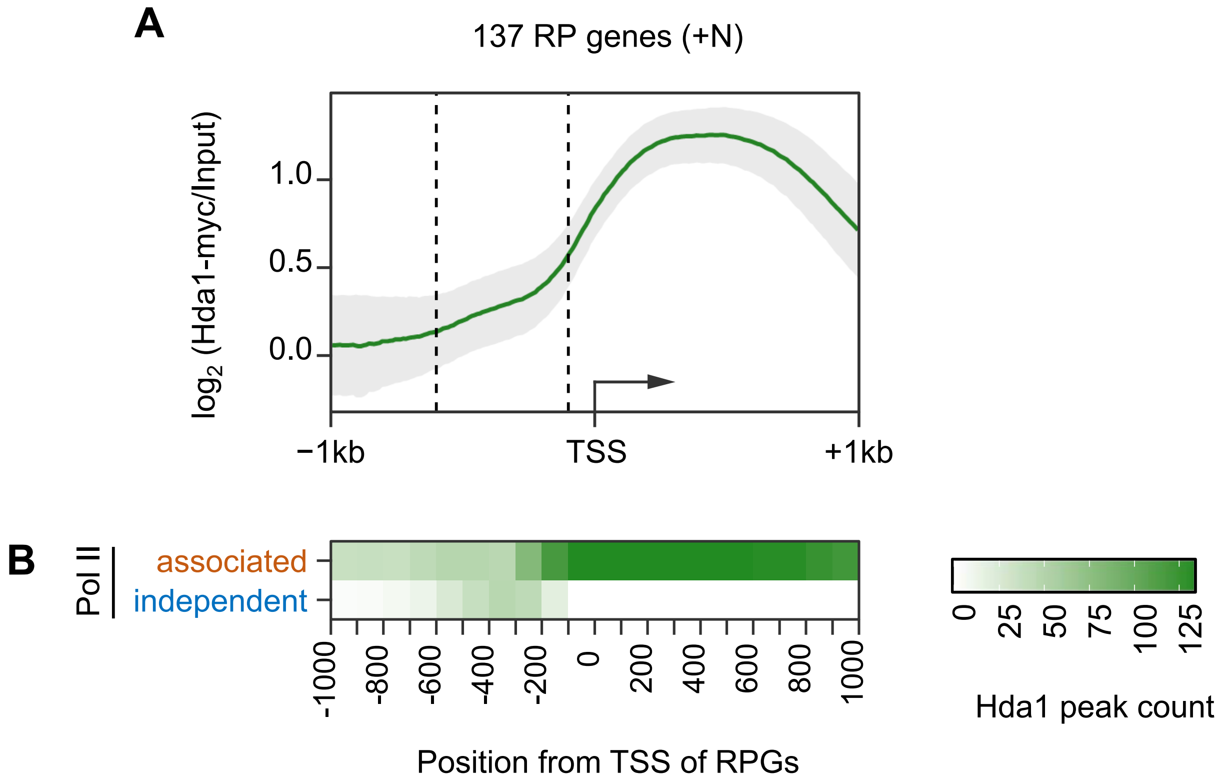

Supplementary Figure S5

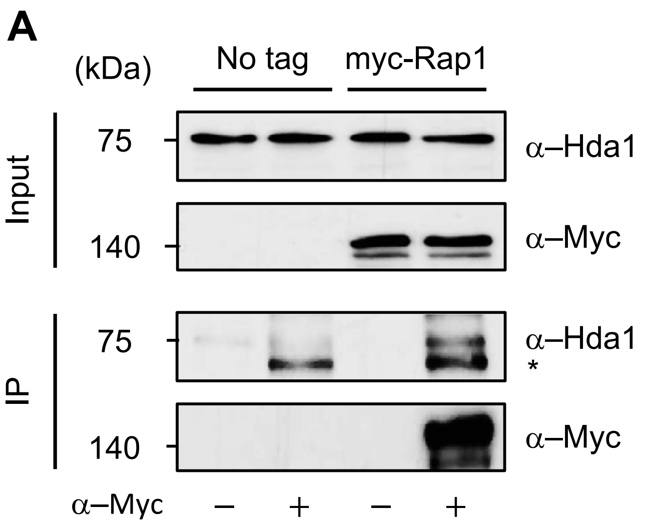

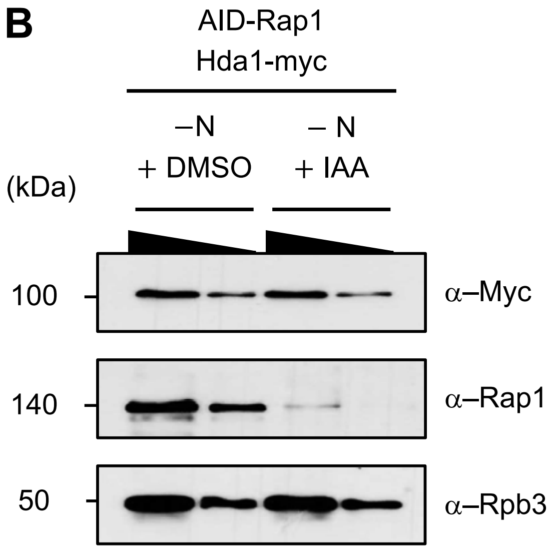

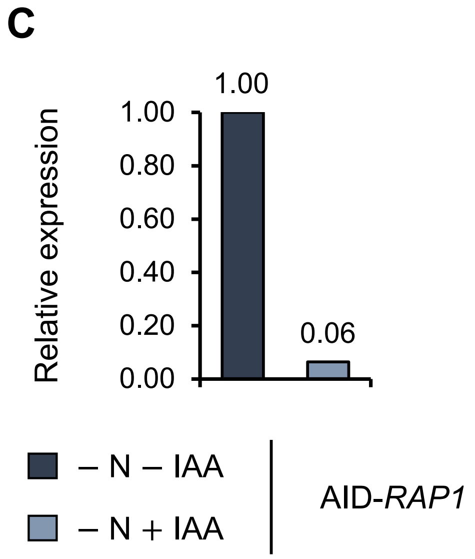

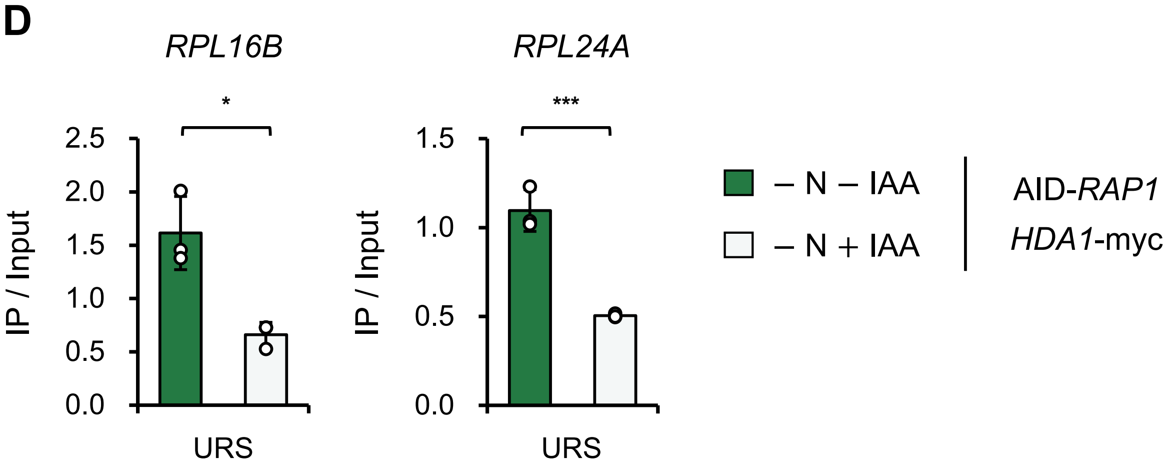

Supplementary Figure S6

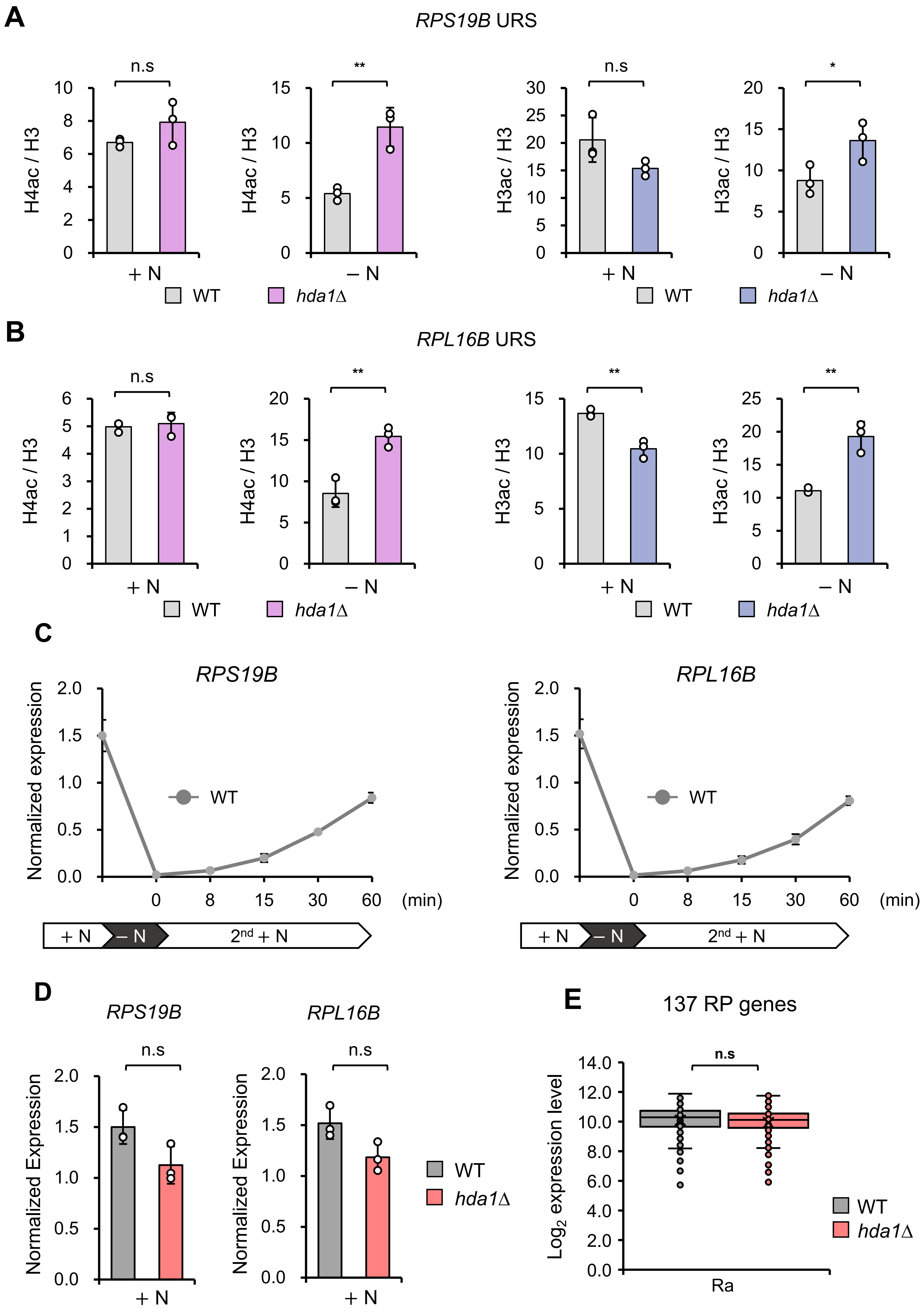

Supplementary Figure S7

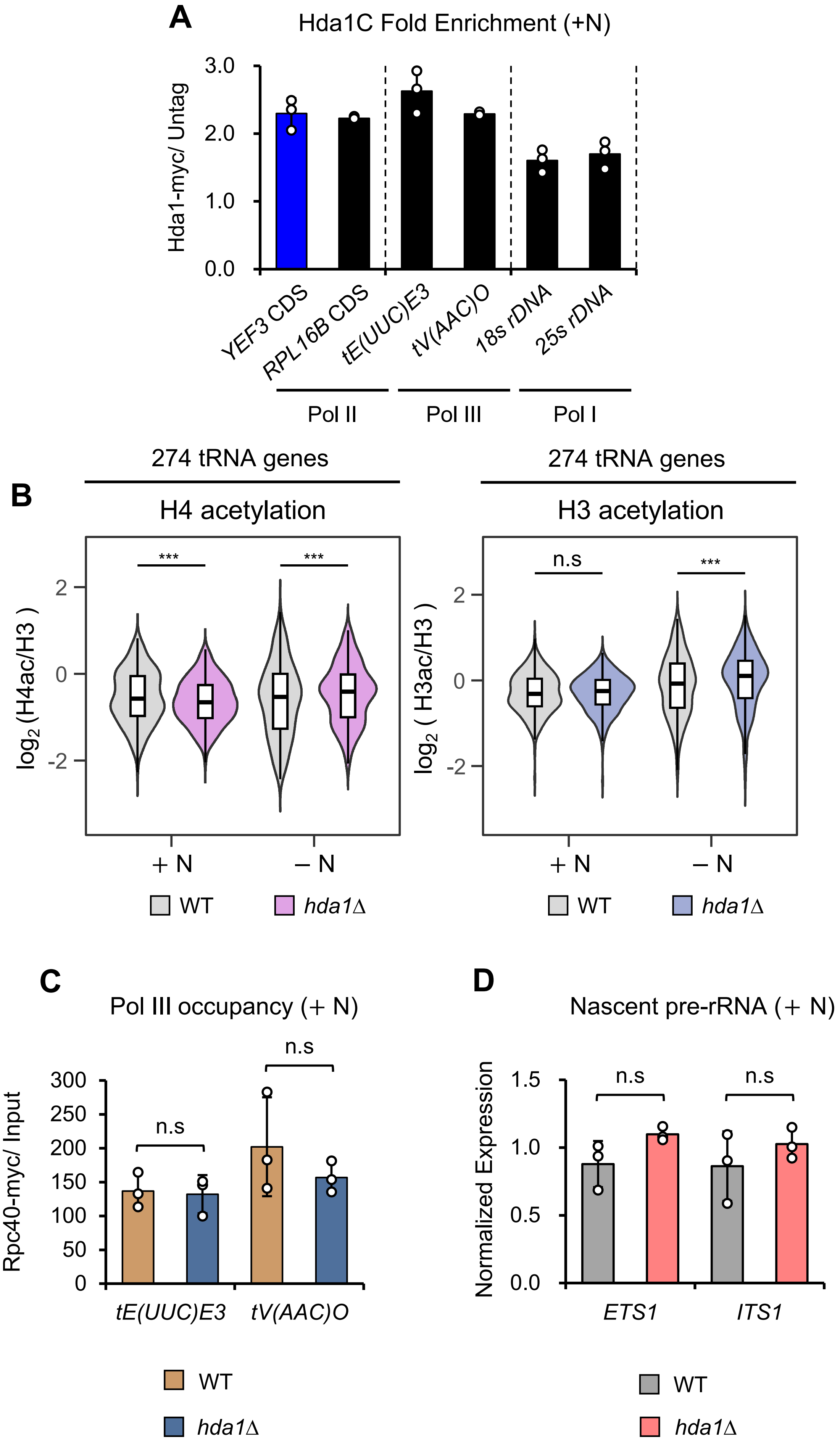

Supplementary Figure S8

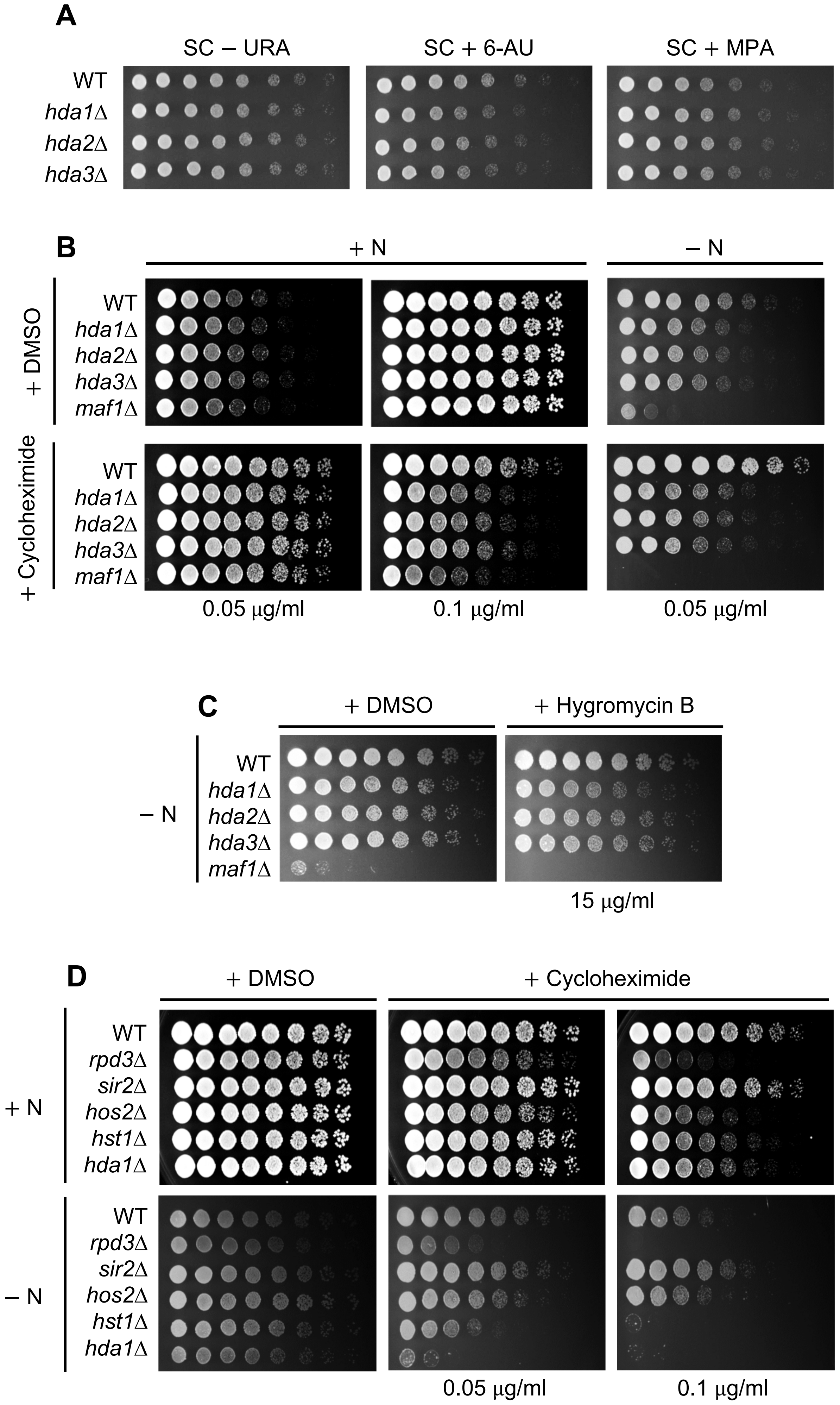

Supplementary Figure S8

E

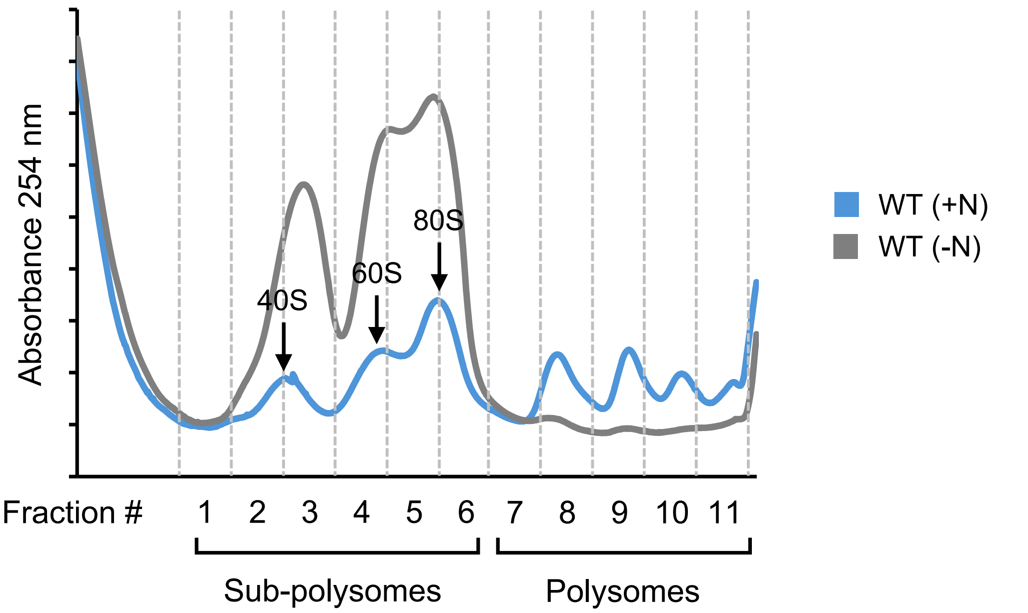

F

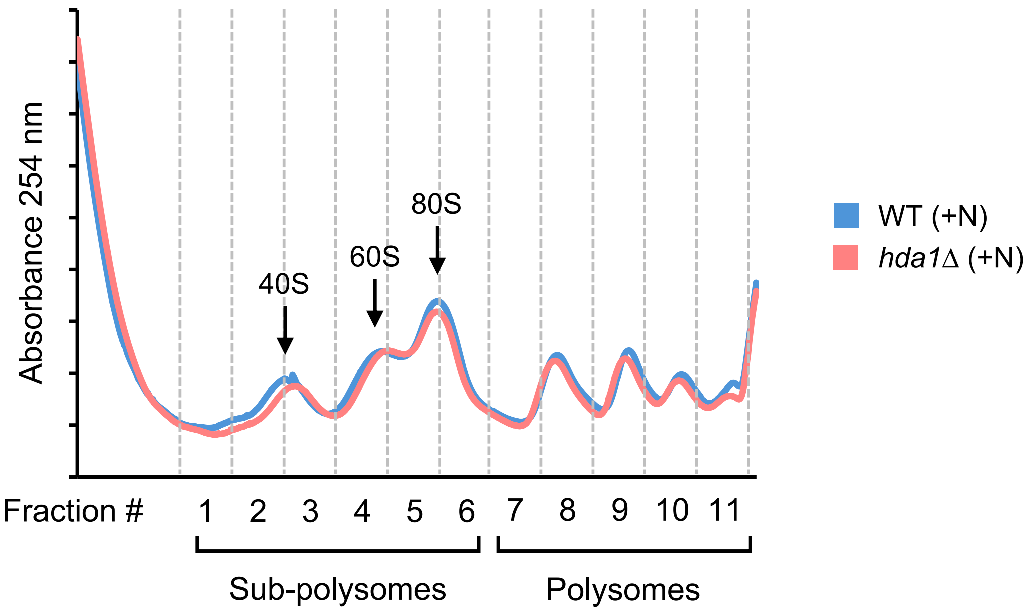

Supplementary Figure S1. Hda1C preferentially deacetylates H4 at active genes and H3 at inactive genes.

(A) Effect of starvation on transcription in *YEF3* and *PMA1*. Rpb3 occupancies were assessed with ChIP-qPCR analysis. The crosslinked chromatin was precipitated with anti-Rpb3 antibody. A non-transcribed region near the telomere of chromosome VI was used as an internal control. The signals for anti-Rpb3 were quantitated and normalized to the input signal. Error bars show the standard deviation (S.D.) calculated from three biological replicates, each with three technical replicates. $^{*}p < 0.05$, $^{**}p < 0.01$, and $^{***}p < 0.001$ (two-tailed unpaired Student's *t* test). **(B)** Correlation heatmap showing the reproducibility of ChIP-seq datasets for Input, Rpb3, and Rpc40 from two independent experiments. **(C)** Correlation heatmap showing the reproducibility of ChIP-seq datasets for Input and Hda1-myc from two independent experiments. **(D)** Cells expressing Hda1-myc were grown in +N and then transferred to -N conditions for 4 hours. Whole cell extracts were subjected to western blot analysis with the indicated antibodies. Rpb3 was used as a loading control. **(E-F)** Heatmaps of H4 and H3 acetylation in +N and -N conditions at the 813 and 1018 genes that are (E) repressed and (F) induced by -N, as determined by ChIP-seq with anti-H4ac (tetra-acetyl) or anti-H3ac (di-acetyl) antibody. The signals for H3 and H4 acetylation were quantitated and normalized to the total H3 content. Acetylation levels are calculated as $\log_2$ fold change in *hda1*Δ cells *versus* wild type cells. The data are from two-independent ChIP-seq experiments. Genes are sorted in descending order their Rpb3 levels in +N (y-axis). The x-axis indicates the position relative to the transcription-start site (TSS) and transcription-end site (TES). **(G)** Correlation heatmap showing the reproducibility of ChIP-seq datasets for H3, H3 acetylation, and H4 acetylation from two independent experiments.

Supplementary Figure S2. RNA Pol II-associated Hda1 binding to coding regions.

(A) Summary of the Pol II-associated and Pol II-independent bins and Hda1 peaks in wild type cells that were grown in +N and -N. **(B-C)** Gene ontology (GO) analysis of the mRNA genes that exhibit Pol II-associated CDS Hda1 peaks in (B) +N and (C) -N conditions. The top 5 enriched terms are shown. **(D)** ChIP-seq tracks of the Rpb3 and Hda1-myc ChIP signals at two 'Intergenic' Hda1 peaks (the Type 2 Hda1 peaks illustrated in Figure 2C).

Supplementary Figure S3. RNA Pol II-independent Hda1 binding to tRNA genes and intergenic regions.

(**A**) ChIP-seq tracks of the Rpb3 and Hda1-myc ChIP signals at tRNA genes that show Pol II-independent peaks (the Type 1 Hda1 peaks illustrated in Figure 2C). **(B)** ChIP-seq tracks of the Rpb3 and Hda1-myc ChIP signals at intergenic regions that have Pol II-independent Hda1 peaks (the Type 3 Hda1 peaks illustrated in Figure 2C).

Supplementary Figure S4. Hda1C strongly binds to coding regions of RP genes in +N conditions.
(A) Line plot showing the average Hda1-myc ChIP-seq signals in the 1kb region flanking the TSS of the 137 RP genes in +N conditions. Standard deviation (S.D.) was indicated in gray. **(B)** Heatmap showing

that the RNA Pol II-associated Hda1 peaks in +N are particularly enriched in coding regions of the 137 RP genes.

Supplementary Figure S5. Hda1C targeting to the URS of RP genes requires Rap1.

(A) Hda1 physically interacts with Rap1 in -N conditions. Whole cell extracts from the indicated strains were incubated with anti-myc antibody and protein G beads. The precipitates (IP) were analyzed by immunoblotting for Hda1 and myc-Rap1. **(B)** Cells expressing AID-Rap1 were treated with IAA for 4h in -N conditions (-N+IAA). As a negative control, the cells were grown in -N with an equal volume of DMSO (-N-IAA). Whole cell extracts were subjected to western blot analysis with the indicated antibodies. Rpb3 was used as a loading control. **(C)** IAA treatment depleted ~94% of the Rap1 proteins. Rap1 signal intensity was quantitated with ImageJ. Values from -N-IAA were set to 1.00, and relative intensity was calculated. **(D)** Depletion of Rap1 reduced Hda1C binding to the URS of RP genes in -N. Crosslinked chromatin was precipitated with anti-myc antibody. ChIP-qPCR analysis of *RPL16B* and *RPL24A* was done as in Figure 4D.

Supplementary Figure S6. Effect of histone deacetylation by Hda1C at RP gene URSs on RP gene expression.

(A-B) Hda1C deacetylates histone H3 and H4 at the URS of RP genes in -N. Wild type and *hda1*Δ cells were grown in +N and -N and the histone acetylation levels at the URSs of (A) *RPS19B* and (B) *RPL16B* were determined by ChIP-qPCR analysis. The crosslinked chromatin from the indicated cells was precipitated with anti-H4ac (tetra-acetyl) or anti-H3ac (di-acetyl) antibody. A non-transcribed region located close to the telomere of chromosome VI was used as an internal control. The signals for H3 and H4 acetylation were quantitated and normalized to the total H3 content. Error bars show the standard deviation (S.D.) calculated from three biological replicates, each with three technical replicates. *$p < 0.05$, **$p < 0.01$, and ***$p < 0.001$ (two-tailed unpaired Student's t test). **(C)** Transcriptional responses of *RPS19B* and *RPL16B* upon nutrient starvation and refeeding. mRNA levels were determined by RT-qPCR with three independent RNA samples. **(D)** Loss of Hda1 has no effect on *RPL16B* and *RPS19B* transcription in +N. mRNA levels were determined by RT-qPCR with three independent RNA samples. **(E)** Hda1 is not required for the basal expression levels of RP genes in raffinose medium. The box plot shows $\log_2$ expression levels of the 137 RP genes in wild type and *hda1*Δ cells.

Supplementary Figure S7. Hda1C binds to RNA Pol I and III-transcribed genes.

(A) Hda1C binds to translation-associated genes that are transcribed by the three RNA polymerases. Crosslinked chromatin from untagged control or *HDA1*-myc grown in +N was precipitated with anti-myc antibody. The anti-myc signals were quantitated and normalized to the input signal. The fold enrichment of Hda1C was calculated by dividing the normalized signals from *HDA1*-myc by those from untagged controls. **(B)** Hda1C deacetylates histone H3 and H4 at tRNA genes in -N. The violin plot shows the average H3 and H4 acetylation profiles in wild type and *hda1*Δ cells. Histone acetylation levels are

quantitated and normalized as shown in Figure 5A. Significance levels were computed by Wilcoxon signed-rank test (*$p < 0.05$, **$p < 0.01$, and ***$p < 0.001$). **(C)** Loss of *HDA1* does not affect RNA Pol III occupancy at tRNA in +N. Crosslinked chromatin from the indicated strains grown in +N was precipitated with anti-myc antibody as shown in Figure 6D. **(D)** *HDA1* deletion does not alter nascent pre-rRNA levels in +N. Nascent pre-rRNA levels were quantitated as described in Figure 6E.

Supplementary Figure S8. Hda1C may affect translation.

(A) Mutants for Hda1C show normal growth in the presence of the transcriptional inhibitors 6-AU and MPA. The indicated cells were spotted in 3-fold dilutions onto synthetic complete (SC) plates lacking Uracil (SC -URA) or SC -URA plates containing 6-AU (150μg/ml) or MPA (40μg/ml). **(B)** Hda1C mutants show severe sensitivity to cycloheximide in -N compared to wild type cells. The indicated cells were spotted on YPD (+N) or 0.15x YP (-N) plates containing DMSO (+N, 1 day or 2 days growth shown/-N, 2 days) or cycloheximide (+N, 2 days/-N, 6 days). **(C)** The indicated cells were spotted on -N plates containing hygromycin B. **(D)** *HDA1*-deleting cells exhibit a more severe growth defect upon cycloheximide treatment in -N than other HDAC deletion mutants. **(E)** Starvation represses global translation. The polysome profiles of wild type cells grown in +N and -N were measured by tracing the UV absorbance at 254 nm (A254) after fractionating the whole cell extracts using a sucrose density gradient. **(F)** Polysome profiles were not changed in wild type and *HDA1* deleting cells in +N.

Supplementary Table S1. Strains used in this study.

Strain	Genotype	Source or Reference
BY4741	MATa, ura3Δ0, leu2Δ0, his3Δ1, met15Δ0	SGD www.yeastgenome.org
YSB787	MATa, bur1Δ::HIS3, ura3-52, leu2Δ1, trp1Δ63, his3Δ200, lys2Δ202 (pRS316-BUR1)	Keogh et al., 2005
YF2239	MATa, his3-Δ200, leu2-3,2-112, lys2-801, trp1-1(am), URA3::TIR-9Myc	Morawska et al., 2013
YTK73	MATa, ura3Δ0, leu2Δ0, his3Δ1, met15Δ0, hst1Δ::KanMX4	This study
YTK75	MATa, ura3Δ0, leu2Δ0, his3Δ1, met15Δ0, hos2Δ::KanMX4	This study
YTK109	MATa, ura3Δ0, leu2Δ0, his3Δ1, met15Δ0, hda1Δ::KanMX4	This study
YTK111	MATa, ura3Δ0, leu2Δ0, his3Δ1, met15Δ0, hda3Δ::KanMX4	This study
YTK113	MATa, bur1Δ::HIS3, ura3-52, leu2Δ1, trp1Δ63, his3Δ200, lys2Δ202 (pRS316-BUR1), hda1Δ::KanMX4	This study
YTK304	MATa, ura3Δ0, leu2Δ0, his3Δ1, met15Δ0, trp1Δ::URA3	This study
YTK376	MATa, ura3Δ0, leu2Δ0, his3Δ1, met15Δ0, trp1Δ::URA3, Hda1-18Myc::TRP1	This study
YTK524	MATa, ura3Δ0, leu2Δ0, his3Δ1, met15Δ0, trp1Δ::URA3, Rpc40-18Myc::TRP1	This study
YTK533	MATa, ura3Δ0, leu2Δ0, his3Δ1, met15Δ0, trp1Δ::URA3, Rpc40-18Myc::TRP1, hda1Δ::KanMX4	This study
YTK573	MATa, ura3Δ0, leu2Δ0, his3Δ1, met15Δ0, maf1Δ::KanMX4	This study
YTK773	MATa, ura3Δ0, leu2Δ0, his3Δ1, met15Δ0, hda2Δ::KanMX4	This study
YTK992	MATa, ura3Δ0, leu2Δ0, his3Δ1, met15Δ0, rpd3Δ::KanMX4	This study
YTK1018	MATa, ura3Δ0, leu2Δ0, his3Δ1, met15Δ0, trp1Δ::URA3, Rap1-18Myc::TRP1	This study
YTK1302	MATa, ura3Δ0, leu2Δ0, his3Δ1, met15Δ0, Hda1-18Myc::HIS3	This study
YTK1455	MATa, ura3Δ0, leu2Δ0, his3Δ1, met15Δ0, trp1Δ::URA3	This study
YTK1535	MATa, his3-Δ200, leu2-3,2-112, lys2-801, trp1-1(am), URA3::TIR-9Myc, pGAL1-AID(IAA17, 71-116)-Rap1::TRP1	This study
YTK1542	MATa, his3-Δ200, leu2-3,2-112, lys2-801, trp1-1(am), URA3::TIR-9Myc, pGAL1-AID(IAA17, 71-116)-Rap1::TRP1, (trp1-pGAL1)Δ::pRAP1	This study
YTK1591	MATa, his3-Δ200, leu2-3,2-112, lys2-801, trp1-1(am), URA3::TIR-9Myc, pGAL1-AID(IAA17, 71-116)-Rap1::TRP1, (trp1-pGAL1)Δ::pRAP1, Hda1-18Myc::TRP1	This study
YTK1683	MATa, ura3Δ0, leu2Δ0, his3Δ1, met15Δ0, sir2Δ::KanMX4	This study
YTK1979	MATa, ura3Δ0, leu2Δ0, his3Δ1, met15Δ0, trp1Δ::URA3, pGAL1-AID(IAA17,71-116)-9Myc-Rap1::TRP1	This study
YTK1997	MATa, ura3Δ0, leu2Δ0, his3Δ1, met15Δ0, trp1Δ::URA3, pGAL1-AID(IAA17,71-116)-9Myc-Rap1::TRP1, (trp1-pGAL1-AID)Δ::pRAP1-ATG	This study
YFP1	MATh- (972)	Daeyeop Lee

Supplementary Table S2. Oligonucleotides used in this study.

Oligo Name	Sequences (5' - 3')	Purpose
YEF3 CDS	(F) GGTTTGAAGTTGAGAAAGTACAAGGG<br>(R) TCAAAGTAGACTTACCAGCACC	ChIP-qPCR
PMA1 CDS	(F) CAGAGTTGTTGAAATCTTGC<br>(R) TGTCTGGAGGTCTTCAAAGC	ChIP-qPCR
RPS19B URS	(F) CCTTTGGTGCACTATTGAT<br>(R) TGCAAAACCTCACCAAACAC	ChIP-qPCR
RPL16B URS	(F) AATTTGGCTGTTTCTCAACG<br>(R) CTGATACAAGGATCAAATGG	ChIP-qPCR
RPL24A URS	(F) GGAAATAAGAGGTTCAACAAG<br>(R) AAATTTAAATGCGGCCCTAG	ChIP-qPCR
RPL16B CDS	(F) CTTTTTGAGAAAGGCTACTG<br>(R) CGAAGATCTTTAAACGTTCC	ChIP-qPCR
RPS19B CDS	(F) AGGTAAATTAGAAGTCCCAG<br>(R) TGCTTTCTCATGTAAATGTG	ChIP-qPCR
tE(UUC)E3	(F) GAGTTGCTTGAATTGTTAACG<br>(R) TTTGATCGTAAGCGATTGAA	ChIP-qPCR
tV(AAC)O	(F) GGTCCGGTTTAGTCCTTTTA<br>(R) TTCTTAAAAACGATTTCGCC	ChIP-qPCR
18s rDNA	(F) GGGGATCGAAGATGATCAGA<br>(R) TTTCTCGTAAGGTGCCGAGT	ChIP-qPCR
25s rDNA	(F) AATCGACCGATCCTGATGTC<br>(R) TACGAGCCTCCACCAGAGTT	ChIP-qPCR
RPS19B	(F) AGGTAAATTAGAAGTCCCAG<br>(R) TGCTTTCTCATGTAAATGTG	Reverse Transcription and qPCR
RPL16B	(F) CTTTTTGAGAAAGGCTACTG<br>(R) CGAAGATCTTTAAACGTTCC	Reverse Transcription and qPCR
ETS1	(F) TGGGTTGATGCGTATTGAGA<br>(R) TCGCTGATTTGAGAGGAGGT	Reverse Transcription and qPCR
ITS1	(F) TGTTTTGGCAAGAGCATGAG<br>(R) TCGAATGCCCAAAGAAAAAG	Reverse Transcription and qPCR
SCR1	(F) GAAGTGTCCCGGCTATAATAAA<br>(R) GACGCTGGATAAAACTCCCC	Reverse Transcription and qPCR
Rap1_18myc	(F) GGTAGAATGGAAATGAGGAAAAGATTTTTTGAGAAGG ACCTGTTATCCGGTTCTGCTGCTAGTGGT<br>(R) AAGGAGTAAAATAAGTTAAACAATGATGTTACTTAATT CAATTACCCTCGAGGCCAGAAGACTAAG	Rap1-Ct-myc
Hda1_18myc	(F) ACAGACTTTATACTGGATTCGTTTGAAGAATGGAGTG ATGAAGAATCCGGTTCTGCTGCTAGTGGT<br>(R) GGCATGAAGGTTGCCGAAAAAAAATTATTAATGGCCA GTTTTTCCCCTCGAGGCCAGAAGACTAAG	Hda1-Ct-myc
Rpc40_18myc	(F) TTAAAGAATAAGGCTGAGTATTTGAAAAACTGTCCAAT TACCCAATCCGGTTCTGCTGCTAGTGGT<br>(R) TATTTTTCTCAAACTGTGTTTTTTTTATTAGTATGCAAA GTAGGACCTCGAGGCCAGAAGACTAAG	Rpc40-Ct-myc

N-term AID_P1_F	(F) GAGCTCGTTTAAACCTCCTT	TRP1-pGAL1-AID cassette
N-term AID_P1_R (Sal I)	(R) ATCGTCGACTAAAGACATTTTGAGATCCG	TRP1-pGAL1-AID cassette
N-term AID_P2_F (Sal I)	(F) ATCGTCGACATGCCTAAAGATCCAGCCAAACC	TRP1-pGAL1-AID cassette
N-term AID_P2_New_R	(R) CTTTTGTTCACCACTAGCCC	TRP1-pGAL1-AID cassette
Rap1_N-term AID_F	(F) TTAAGAAGGGGCAATATGACTATGAATTAATTCAAGTA CAATGCTCTTAAGAGCTCGTTTAAACCTCCTT	pGAL1-Nt-AID-Rap1
Rap1_N-term AID_R	(R) AATGCATCAACATATTCTGCTGGTGCAGTTTCAAAAT CATCTGGACTAGATGATACCTTCACGAACGCC	pGAL1-Nt-AID-Rap1
Rap1 prom (Up209)_F	(F) ACACACTGTGGGACAAATAC	pRAP1-Nt-AID or 9Myc-Rap1
Rap1 prom exchange_R	(R) GGCCATCCCACAACTTGTGCCTTGGCCGGAGGTTTG GCTGGATCTTTAGGCATATTGAGATAATCTGTACGCA	pRAP1-Nt-AID-Rap1
Rap1 prom exchange_R_New	(R) CAACTTTTGTTCACCACTAGCCCCGGGTGATACCTTC ACGAA CGCCATATTGAGATAATCTGTACGCA	pRap1-Nt-9Myc-Rap1
Hda1_F	(F) TAGGTAAATAGAGCTGGGAG	Knockout
Hda1_R	(R) GTAAAACACAGCTTAATGCATT	Knockout
Hda2_KanMX4_F	(F) GGCTTCATTAGTGTGTGAAAAATAAAGAAAATAGATA CAATACTATCGACAGATTGTACTGAGAGTGCAC	Knockout
Hda2_KanMX4_R	(R) ATAAAATCTCTCTATATTATACAGGCTACTTCTTTTAG GAAACGTCACATCTGTGCGGTATTTCACACCG	Knockout
Hda2_F	(F) TAAAATAACATAATTGCGGCAC	knockout
Hda2_R	(R) GTTGATGAAATGTTTCTGAAGA	Knockout
Hda3_F	(F) TACTGATTTAATCCACTCAGTT	Knockout
Hda3_R	(R) TGTATAGTAGTGATACGTGGT	Knockout
Maf1_F	(F) GCAATGCGAAAAATATAGGC	Knockout
Maf1_R	(R) CCTCGCTAAAGTGTTCAATT	Knockout
Rpd3_F	(F) ATATGTCCCATATTTTGCCT	Knockout
Rpd3_R	(R) GGCAATTTTCTTCGAAACGT	Knockout
Sir2_F	(F) CGAGGAATTTTTCTTTGTCCT	Knockout
Sir2_R	(R) AAGCTATTTGTGAGAGCCTT	Knockout
Hos2_F	(F) ACTCTATATAGAGGATTCTCGAT	Knockout
Hos2_R	(R) TGCACCTTCCAAAAATGTTT	Knockout
Hst1_F	(F) TGGCCGCAACAAATGAT	Knockout
Hst1_R	(R) ATTAAATGATGAAATTAAACAA	Knockout
